# Annexin A1 expression in a pooled breast cancer series: association with tumor subtypes and prognosis

**DOI:** 10.1186/s12916-015-0392-6

**Published:** 2015-07-02

**Authors:** Marcelo Sobral-Leite, Jelle Wesseling, Vincent T. H. B. M. Smit, Heli Nevanlinna, Martine H. van Miltenburg, Joyce Sanders, Ingrid Hofland, Fiona M. Blows, Penny Coulson, Gazinska Patrycja, Jan H. M. Schellens, Rainer Fagerholm, Päivi Heikkilä, Kristiina Aittomäki, Carl Blomqvist, Elena Provenzano, Hamid Raza Ali, Jonine Figueroa, Mark Sherman, Jolanta Lissowska, Arto Mannermaa, Vesa Kataja, Veli-Matti Kosma, Jaana M. Hartikainen, Kelly-Anne Phillips, kConFab/AOCS Investigators, Fergus J. Couch, Janet E. Olson, Celine Vachon, Daniel Visscher, Hermann Brenner, Katja Butterbach, Volker Arndt, Bernd Holleczek, Maartje J. Hooning, Antoinette Hollestelle, John W. M. Martens, Carolien H. M. van Deurzen, Bob van de Water, Annegien Broeks, Jenny Chang-Claude, Georgia Chenevix-Trench, Douglas F. Easton, Paul D. P. Pharoah, Montserrat García-Closas, Marjo de Graauw, Marjanka K. Schmidt

**Affiliations:** Division of Molecular Pathology, Netherlands Cancer Institute, Amsterdam, The Netherlands; Programa de Farmacologia, Instituto Nacional do Câncer (INCA), Rio de Janeiro, RJ Brazil; Division of Diagnostic Oncology, Netherlands Cancer Institute, Amsterdam, The Netherlands; Department of Pathology, Leiden University Medical Center, Leiden, The Netherlands; University of Helsinki, Helsinki, Finland; Department of Obstetrics and Gynecology, Helsinki University Central Hospital, Helsinki, Finland; Core Facility Molecular Pathology and Biobanking, Division of Molecular Pathology, Netherlands Cancer Institute, Amsterdam, The Netherlands; Centre for Cancer Genetic Epidemiology, Department of Oncology, University of Cambridge, Cambridge, UK; Division of Genetics and Epidemiology, Institute of Cancer Research, London, UK; Breakthrough Breast Cancer Centre, London, UK; Department of Pharmacoepidemiology & Clinical Pharmacology, Utrecht Institute of Pharmaceutical Sciences (UIPS), Utrecht, The Netherlands; Department of Pathology, Helsinki University Central Hospital, Helsinki, Finland; Department of Clinical Genetics, Helsinki University Central Hospital, Helsinki, Finland; Department of Oncology, Helsinki University Central Hospital, Helsinki, Finland; Cancer Research UK Cambridge Institute Oncology, University of Cambridge, Cambridge, UK; Department of Histopathology, Addenbrooke’s Hospital, Cambridge University Hospital NHS Foundation Trust, Cambridge, UK; Department of Pathology, University of Cambridge, Cambridge, UK; Division of Cancer Epidemiology and Genetics, National Cancer Institute, Rockville, MD USA; Division of Cancer Prevention, National Cancer Institute, Rockville, MD USA; Department of Cancer Epidemiology and Prevention, Maria Sklodowska-Curie Memorial Cancer Center and Institute of Oncology, Warsaw, Poland; School of Medicine, Institute of Clinical Medicine, Pathology and Forensic Medicine, University of Eastern Finland, Kuopio, Finland; Cancer Center of Eastern Finland, University of Eastern Finland, Kuopio, Finland; Imaging Center, Department of Clinical Pathology, Kuopio University Hospital, Kuopio, Finland; Cancer Center, Kuopio University Hospital, Kuopio, Finland; Jyväskylä Central Hospital, Jyväskylä, Finland; Division of Cancer Medicine, Peter MacCallum Cancer Centre, Melbourne, Australia; Sir Peter MacCallum Department of Oncology, The University of Melbourne, Melbourne, Australia; Centre for Molecular, Environmental, Genetic and Analytic Epidemiology, School of Population Health, The University of Melbourne, Melbourne, Australia; Department of Medicine, St Vincent’s Hospital, The University of Melbourne, Melbourne, Australia; Department of Laboratory Medicine and Pathology, Mayo Clinic, Rochester, MN USA; Department of Health Sciences Research, Mayo Clinic, Rochester, MN USA; Division of Clinical Epidemiology and Aging Research, German Cancer Research Center (DKFZ), Heidelberg, Germany; German Cancer Consortium (DKTK), German Cancer Research Center (DKFZ), Heidelberg, Germany; Division of Preventive Oncology, German Cancer Research Center (DKFZ), Heidelberg, Germany; Saarland Cancer Registry, Saarbrücken, Germany; Department of Medical Oncology, Erasmus MC Cancer Institute, Rotterdam, The Netherlands; Department of Pathology, Erasmus MC Cancer Institute, Rotterdam, The Netherlands; Division of Toxicology, Leiden Academic Centre for Drug Research, Leiden University, Leiden, The Netherlands; Division of Cancer Epidemiology, Unit of Genetic Epidemiology, German Cancer Research Center (DKFZ), Heidelberg, Germany; Department of Genetics, QIMR Berghofer Medical Research Institute, Brisbane, Australia; Division of Psychosocial Research and Epidemiology, Netherlands Cancer Institute, Plesmanlaan 121, 1066, CX Amsterdam, The Netherlands

**Keywords:** Breast cancer, Annexin A1, *BRCA1* and *BRCA2* mutations

## Abstract

**Background:**

Annexin A1 (ANXA1) is a protein related with the carcinogenesis process and metastasis formation in many tumors. However, little is known about the prognostic value of ANXA1 in breast cancer. The purpose of this study is to evaluate the association between ANXA1 expression, *BRCA1/2* germline carriership, specific tumor subtypes and survival in breast cancer patients.

**Methods:**

Clinical-pathological information and follow-up data were collected from nine breast cancer studies from the Breast Cancer Association Consortium (BCAC) (n = 5,752) and from one study of familial breast cancer patients with *BRCA1/2* mutations (n = 107). ANXA1 expression was scored based on the percentage of immunohistochemical staining in tumor cells. Survival analyses were performed using a multivariable Cox model.

**Results:**

The frequency of ANXA1 positive tumors was higher in familial breast cancer patients with *BRCA1/2* mutations than in BCAC patients, with 48.6 % versus 12.4 %, respectively; *P* <0.0001. ANXA1 was also highly expressed in BCAC tumors that were poorly differentiated, triple negative, EGFR-CK5/6 positive or had developed in patients at a young age. In the first 5 years of follow-up, patients with ANXA1 positive tumors had a worse breast cancer-specific survival (BCSS) than ANXA1 negative (HR_adj_ = 1.35; 95 % CI = 1.05–1.73), but the association weakened after 10 years (HR_adj_ = 1.13; 95 % CI = 0.91–1.40). ANXA1 was a significant independent predictor of survival in HER2+ patients (10-years BCSS: HR_adj_ = 1.70; 95 % CI = 1.17–2.45).

**Conclusions:**

ANXA1 is overexpressed in familial breast cancer patients with *BRCA1/2* mutations and correlated with poor prognosis features: triple negative and poorly differentiated tumors. ANXA1 might be a biomarker candidate for breast cancer survival prediction in high risk groups such as HER2+ cases.

**Electronic supplementary material:**

The online version of this article (doi:10.1186/s12916-015-0392-6) contains supplementary material, which is available to authorized users.

## Background

Breast cancer is a heterogeneous group of pathologic entities with different risk of recurrence and therapy response [1]. In order to improve breast cancer diagnosis and treatment decision, it is necessary to gain a better understanding of the metastatic pathways and etiology.

Annexin A1 (ANXA1) protein binds the cellular membrane phospholipids in a Ca^2+^ regulated manner and can be phosphorylated on several residues both on the N-terminal functional domain and on the C-terminus core [2] by different proteins, such as the epidermal growth factor receptor (EGFR) [3], insulin receptor (IR) [4], TRPM7 channel kinase 1 (ChaK1) [5], protein kinase C (PKC) and protein kinase A (PKA) [6]. ANXA1 has been found in several tissues and regulates physiological mechanisms such as hormone secretion [7], EGFR degradation [8], membrane transport [9], apoptosis [10] and cell differentiation [11]. As a glucocorticoid-induced molecule, ANXA1 plays an important role in the inflammatory response [12].

ANXA1 expression is related with the carcinogenesis process [13–15] and with metastasis formation in many tumors [16–18], including breast tumors [19–23], where we and others have shown that ANXA1 overexpression is associated with high pathological differentiation grade, lack of hormone receptor expression and a basal-like phenotype [20, 24, 25]. Patients with *BRCA1* or *BRCA2* (*BRCA1/2*) germline mutations often present tumors with these characteristics, but until now there are no data in the literature implicating a link between high ANXA1 expression and familial breast cancer. The main focus of this study was to analyze the relationship between high ANXA1 tumor expression with *BRCA1/2* germline carriership and survival in breast cancer patients, including those with specific tumor subtypes, using a large dataset of pooled breast cancer series. These analyses allow us to explore the potential of ANXA1 as a marker for breast cancer outcome prediction and treatment response.

## Methods

### Study populations

The international Breast Cancer Association Consortium (BCAC) comprises a large number of studies investigating the role of common germline genetic variation in breast cancer susceptibility [26]. Nine studies from Europe, North America, New Zealand and Australia contributed with 8,182 cases to this ANXA1 study (Additional file 1: Table S1). All studies were approved by the relevant ethics committees and informed consent was obtained from all participants (Additional file 1: Table S1). Clinical-pathological information and follow-up data were collected by each study individually through medical records, cancer registries and cause of death registries. Data were pooled in the BCAC database according to a data dictionary, and centrally checked for accuracy and consistency. Data included were: age at diagnosis; behavior (*in situ* or invasive); morphology (ductal, lobular and others); tumor size (≤2 cm, >2 and ≤5 cm, or >5 cm); differentiation grade (1, 2 or 3); lymph node status (negative or positive); and breast cancer treatment (radiotherapy, hormonal therapy and chemotherapy). The most common source of data for ER, PR and HER2 status was from medical records, followed by immunohistochemistry (IHC) performed on tumor tissue microarrays (TMAs) or whole section tumor slides. The subtypes were defined as follows: luminal 1 (ER+ and/or PR+ and HER2-); luminal 2 (ER+ and/or PR+ and HER2+); HER2-like (ER-, PR- and HER2+); and triple negative (ER-, PR- and HER2-). Data on CK5/6 and EGFR tumor status were derived from IHC performed on TMAs or whole sections detailed previously [27]. The p53 staining data (received only from one breast cancer study) and the *BRCA1* and *BRCA2* status mutation of the BCAC patients were obtained as described previously [28, 29]. A specific cohort of 132 *BRCA1/2* mutated (BRCA1|2), familial breast cancer patients (a minimum of three first- or second-degree relatives affected with breast and/or ovarian cancer in a family) were included from the Helsinki University Central Hospital (HUCH) in southern Finland as described previously [30]. In addition, within the BCAC there were a few cases from some studies known to be *BRCA1/2* mutated and we excluded these for the analyses comparing BCAC with BRCA1|2 tumors.

### ANXA1 staining

Ninety TMA slides from 8,705 patients were received for ANXA1 staining (including 1 to 6 tumor cores per patient). The ANXA1 staining was performed at the Core Facility Molecular Pathology and Biobanking (CFMPB) at the NKI-AVL on a BenchMark ULTRA autostainer (Ventana Medical Systems, Tucson, AZ, USA). Briefly, paraffin sections were heated at 75 °C for 28 min and deparaffinized in the instrument with EZ Prep solution (Ventana Medical Systems). Heat-induced antigen retrieval was carried out using Cell Conditioning 1 (CC1; Ventana Medical Systems) for 64 min at 95 °C. ANXA1 was detected by incubating sections with antibody clone 29/Annexin I (610066; BD Transduction Laboratories, Franklin Lakes, NJ, USA), 1/1500 dilution for 1 h. Specific reactions were detected using ultraView Universal DAB Detection Kit (Ventana Medical Systems) and slides were counterstained with hematoxylin.

The ANXA1 expression in tumor cells was scored only if the spot was evaluable, subjectively defined as approximately more than 30 % of tumor cells present in the spot. ANXA1 is also expressed in stromal cells but this was not included in the evaluation. Three variables were evaluated: intensity of the staining (negative, weak, moderate or strong); cellular location of the staining (cytoplasm and nucleus or only in cytoplasm); and the percentage of stained cells (0 % to 100 %). Scores were categorized as: 0, no expression; 1+, 10 % to 30 % stained; 2+, 40 % to 70 %; and 3+, 80 % or more cells stained (Additional file 2: Figure S1). The intensity of the staining and the cellular location scores did not contribute to the discernment of different groups by ANXA1 stains and therefore were not further used in the analysis (Additional file 3: Figures S2A and S2B).

Evaluation of ANXA1 expression levels were performed by MdG with consultation of three pathologists (JW, JS and VTS). The concordance was around 81.7 % and 92.4 % among all of them, considering 0 or 1+ as negative and 2+ or 3+ as positive, with a kappa value of 0.86, considering positive versus negative cases between MdG and JW. A subset of 452 patients in the previously published ABCS study were rescored for this study [24].

Around 20 % (n = 2,124) of the included patients enrolled in TMA constructions could not be scored due to technical problems (no sample or less than 30 % of tumor cells in the spot), but the clinical-pathological variable distributions did not differ between patients with or without ANXA1 scores (Additional file 4: Table S2). For analyses, we clustered 0 and 1+ groups based on previous experience [24]. Of note, overall survival (OS) and breast cancer-specific survival (BCSS) did not differ between the 0 and 1+ groups nor between the 2+ and 3+ groups (Additional file 3: Figures S2C and S2D).

### Statistical analyses

In total, we included 6,177 patients for descriptive analysis. For association and survival analysis, the *in situ* breast cancer cases were excluded (n = 204). Patients diagnosed with distant metastases at diagnosis of the primary tumor (n = 31) and those who received chemotherapy before the surgery (n = 84) were also excluded (Additional file 5: Figure S3).

**Fig. 1:**
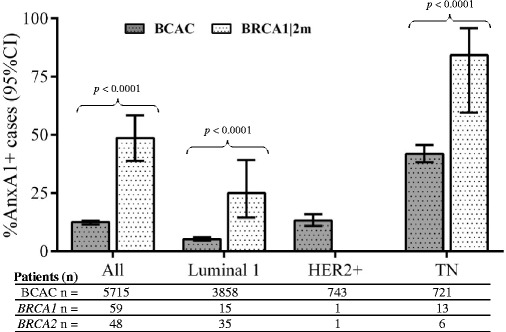
ANXA1 expression profile. Percentage of patients with ANXA1 positive tumors according to breast cancer subtypes comparing BCAC (excluding 37 patients with *BRCA1* or *BRCA2* mutations) versus *BRCA1/2* mutation carriers. For the subtype analysis, patients with missing information for ER, PR and/or HER2 were excluded (393 in BCAC and 36 in *BRCA1/2* mutation carriers). Luminal 1 subtype was defined as ER+ and/or PR+ and HER2-, and triple negative (TN) was defined as ER-, PR- and HER2-. Numbers of HER2+ were too small in the *BRCA1/2* mutation carriers to make a comparison. ANXA1: Annexin A1; BCAC: Breast Cancer Association Consortium; TN: Triple negative

In the case of significant associations between ANXA1 expression and a histopathological variable as evaluated by the Chi-square test, the odds ratios (OR) and their respective 95 % confidence intervals (95 % CI) adjusted for independent clinical variables (OR_adj_) were assessed using logistic regression models. The ANXA1 expression was tested for linear-by-linear associations to calculate trend significances (P_trend_) between tumor subtypes in Fig. 1. The statistical association analyses were conducted using SPSS 20 (SPSS Inc., Chicago, IL, USA).

Survival time was calculated from date of diagnosis to date of death/censoring. In order to allow for prevalent cases, time at risk was calculated from date of study entry to date of death/censoring. This generates an unbiased estimate of the hazard ratio (HR) provided the Cox proportional hazards assumption holds [31]. End of follow-up was defined as the date of (breast cancer) death, last follow-up or 10 years, whichever came first. Distant metastasis-free survival (DMFS) analysis was performed as well, with time censored at 5 years. HRs were estimated using Cox regression models, stratified by BCAC studies. Multivariate Cox models were fitted including the variables associated with breast cancer prognosis: age at diagnosis as continuous variable; tumor grade (1, 2 or 3); tumor size (≤2 cm, >2 cm and ≤5 cm, or >5 cm); lymph node status (negative versus positive); ER/PR status (ER and PR negative versus ER or PR positive); and HER2 receptors status (negative versus positive) as categorical covariates. The analyses were performed as a complete case analysis and a secondary analysis was performed including the missing values in the model. ER/PR status was included as a time-varying covariate because of violation of the proportional hazards assumption using the Schoenfeld residuals test in the multivariable model (*P* <0.0001). Adjustment for chemotherapy and/or hormonal therapy did not significantly change the results and these were not included in the final models. All *P* values reported are from two-sided tests and the threshold for significance was set at *P* = 0.05. The survival analyses were performed using STATA version 11.0 (StataCorp, TX, USA).

## Results

Analyses included 5,752 patients from BCAC cohorts, including cases not known to be *BRCA1/2* carriers, and 107 breast cancer patients from one study of familial breast cancer patients with *BRCA1/2* mutations (Table 1; Additional file 4: Table S2; Additional file 5: Figure S3).

**Table 1:** ANXA1 expression and clinical variables

	BCAC patients					BRCA1|2 mutation carriers					
	ANXA1 negative		ANXA1 positive			ANXA1 negative		ANXA1 positive			
	n	%	n	%	*P* value^a^	n	%	n	%	*P* value^b^	*P* value^c^
	5,040	87.6	712	12.4		55	51.4	52	48.6		<0.0001
Age of diagnosis					<0.0001					0.2253	0.0401
<50 years old	2,462	85.6	413	14.4		34	47.2	38	52.8		
≥50 years old	2,578	89.6	299	10.4		21	60.0	14	40.0		
Missing	0		0			0		0			
*BRCA* status					0.0026					0.0583	
Non-carrier	858	83.6	168	16.4							
*BRCA1*	11	61.1	7	38.9		25	42.4	34	57.6		
*BRCA2*	14	73.7	5	26.3		30	62.5	18	37.5		
Missing	3,812		430			0		0			
Menopausal status					0.0330						
Pre-	1,556	86.2	250	13.8							
Post-	2,258	88.3	298	11.7							
Missing	1,226		164								
Morphology					<0.0001					0.5946	0.0235
Ductal	3,558	86.2	568	13.8		33	50.8	32	49.2		
Lobular	710	94.3	43	5.7		11	61.1	7	38.9		
Missing	772		101			11		13			
Grade					<0.0001					0.0040	0.1460
1	1,129	95.0	59	5.0		7	77.8	2	22.2		
2	2,246	92.8	173	7.2		26	66.7	13	33.3		
3	1,306	75.2	431	24.8		19	35.8	34	64.2		
Missing	359		49			3		3			
Tumor size					<0.0001					0.9910	0.7780
≤2 cm	2,809	88.8	354	11.2		31	51.7	29	48.3		
>2 cm and ≤5 cm	1,610	86.5	252	13.5		19	52.8	17	47.2		
>5 cm	109	79.9	29	21.0		2	50.0	2	50		
Missing	512		77			3		4			
Node status					0.2725						
Negative	2,669	87.1	395	12.9		31	47.0	35	53.0	0.3044	0.1790
Positive	1,955	88.1	263	11.9		22	59.5	15	40.5		
Missing	416		54			2		2			
ER status					<0.0001						
Negative	977	67.8	465	32.2		13	28.3	33	71.7	<0.0001	0.7373
Positive	3,796	95.0	200	5.0		36	75.0	12	25.0		
Missing	267		47			6		7			
PR status					<0.0001						
Negative	1,435	75.9	455	24.1		20	35.1	37	64.9	<0.0001	0.1670
Positive	3,159	94.8	175	5.2		27	77.1	8	22.9		
Missing	446		82			8		7			
HER2 status					0.1328						
Negative	3,484	88.7	442	11.3		25	48.1	27	51.9	1.0000	0.0684
Positive	643	86.8	98	13.2		1	50.0	1	50.0		
Missing	913		172			29		24			
EGFR-CK5/6 status^d^					<0.0001						
Negative	3,317	93.8	218	6.2							
Positive	541	67.6	259	32.4							
Missing	1,182		235								
p53 status^e^					0.0572						
Negative	172	90.1	19	9.9		17	45.9	20	54.1	0.0211	0.8116
Positive	57	80.3	14	19.7		3	14.3	18	85.7		
Missing	596		89			35		14			
Adjuvant chemotherapy					<0.0001						
No	2,593	89.9	282	10.1							
Yes	1,585	83.2	320	16.8							
Missing	862		100								
Adjuvant hormonal therapy					<0.0001						
No	2,059	82.5	438	17.5							
Yes	2,562	92.6	204	7.4							
Missing	419		70								

Distribution of the clinical variables in breast cancer patients according to the ANXA1 expression in all invasive tumors from the BCAC and BRCA1|2 set of patients

*ANXA1* annexin A1, *BCAC* Breast Cancer Association Consortium, *EGFR* epidermal growth factor receptor

^a^ *P* value of the comparison between ANXA1 positive and negative patients in the BCAC set

^b^ *P* value of the comparison between ANXA1 positive and negative patients in the BRCA1|2 mutated set

^c^ *P* value of the comparison between the two sets of ANXA1 positive patients: BCAC and BRCA1|2 mutated patients

^d^EGFR-CK5/6 status was defined as positive when CK5/6 and/or EGFR were positive

^e^in the BCAC set, p53 status information was only available for the ABCS study

### ANXA1 expression in breast cancer patients

The distribution of patients and tumor characteristics of BCAC and *BRCA1/2* mutated patients is shown in Table 1. Younger patients (<50 years old) had slightly more often ANXA1 positive tumors compared with the older group (≥50 years old), in both subsets of patients. Poorly differentiated (high grade) tumors were associated with ANXA1 positive expression (tumor grade 2 or 3: OR_adj_ = 1.59; 95 % CI = 1.04–2.43); as well as those positive for basal-like markers (EGFR and/or CK5/6 positive) or triple negative (OR_adj_ = 4.21; 95 % CI = 3.22–5.50 and OR_adj_ = 6.01; 95 % CI = 4.61–8.01, respectively) (Additional file 6: Table S3).

ANXA1 expression was higher in the tumors from *BRCA1/2* mutated patients compared to BCAC patients overall: 48.6 % versus 12.4 %, respectively; *P* <0.0001, and within specific breast cancer subtypes (Fig. 1). Although all *BRCA1/2* mutated carriers were only from Finland, the proportion of ANXA1 positives in the Finnish BCAC study (HEBCS) was the same compared to that of other BCAC studies (Additional file 1: Table S1A). Triple negative tumors in *BRCA1/2* mutated carriers showed a higher ANXA1 expression than triple negative breast cancer patients in the BCAC cohort (84.2 % versus 41.9 %, respectively; *P* <0.0001). Also, there was a trend for higher ANXA1 expression with a decrease in hormone receptor positivity (i.e. subtypes ranging from luminal to HER2+ to triple negative; P_trend_ <0.0001) (Fig. 1). Moreover, the *BRCA1* patients presented a slightly higher expression of ANXA1 compared with *BRCA2* mutated patients (57.6 % versus 37.5 %, respectively; *P* = 0.0583) (Table 1). In *BRCA1/2* mutated patients, ANXA1 expression was associated with p53 positive status (OR_adj_ = 14.97; 95 % CI = 1.38–163.49; Additional file 6: Table S3).

### Survival analysis according to ANXA1 expression

**Fig. 2:**
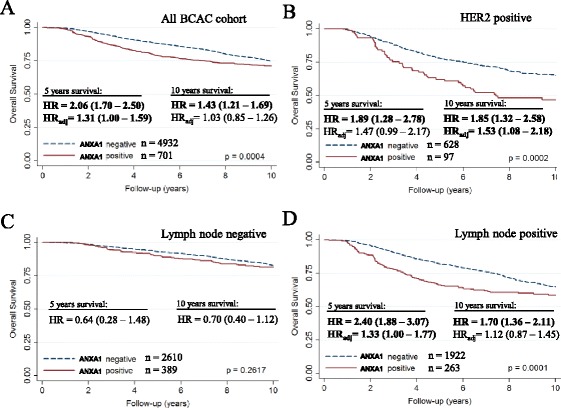
Survival analysis. Survival curves, crude hazard ratios (HR) and adjusted hazard ratios (HR_adj_) in patients from BCAC according to ANXA1 expression for overall survival in (**a**) all BCAC patients, (**b**) HER2 positive patients, (**c**) lymph node negative patients and (**d**) lymph node positive patients. Hazard ratios were adjusted for: age of diagnosis; tumor grade; tumor size; lymph node metastasis; ER/PR status; and HER2 status. ANXA1: Annexin A1; BCAC: Breast Cancer Association Consortium; HR: Hazard ratio

We performed survival analysis of all BCAC patients with follow-up information (follow-up mean: 8.9 years). Patients with ANXA1 positive tumors showed a worse survival than the ANXA1 negative ones, both for OS (Fig. 2a; *P* = 0.0004) and BCSS (Additional file 7: Figure S4A; *P* <0.0001). Similar trends of worse survival were seen in the nine separate cohorts, except for MCBCS (data not shown). After adjustment for clinical variables, a significant association between ANXA1 positivity and worse survival was observed only during the first 5 years of follow-up, but not after 10 years of follow-up (5-years BCSS: HR_adj_ = 1.35; 95 % CI = 1.05–1.73 and 10-years BCSS: HR_adj_ = 1.13; 95 % CI = 0.91–1.40; see also Additional file 8: Table S4). The strong association of ANXA1 expression with poorly differentiated grades and triple negative status likely contributed to this lack of association after 5 years. Similar time-dependent differences in survival were observed in lymph node positive patients (Fig. 2d and Additional file 7: Figure S4D), but ANXA1 expression did not influence survival in lymph node negative patients (Fig. 2c and Additional file 7: Figure S4C).

Evaluating the tumor subtypes, ANXA1 high expression was specifically associated with an increased mortality in HER2 positive patients (10-years OS: HR_adj_ = 1.60; 95 % CI = 1.06–2.41 and 10-years BCSS: HR_adj_ = 1.70; 95 % CI = 1.17–2.45; Fig. 2b and Additional file 7: Figure S4B). The *P* values for interaction between ANXA1 and HER2 in a full model for BCSS or OS, including HER2 positive and negative cases, were 0.136 and 0.140, respectively. In addition, ANXA1 positive cases showed a slightly worse survival in the subgroup of patients older than 49 years old, which seemed to be related to menopausal status (data not shown). For none of the subgroup analyses the DMFS was significantly different between ANXA1 high and low expression groups.

### ANXA1 expression and treatment response

In order to explore the value of the ANXA1 expression in therapy resistance, we performed survival analyses in the group of patients who received adjuvant chemotherapy, as currently recommended. Using clinical guidelines [32], the BCAC patients were classified according to the risk of recurrence using classic prognostic factors for determining the chance of distant metastases occurrence. Within the group that received adjuvant chemotherapy, patients with high risk of recurrence (HER2+ and/or pN+) showed a slightly worse OS and BCSS when ANXA1 was positive, compared with the ANXA1 negative ones in the group aged 50–69 years only (BCSS HR_adj_ = 2.02; 95 % CI = 1.21–3.36 and HR_adj_ = 1.41; 95 % CI = 0.80–2.50; Additional file 9: Figure S5).

**Fig. 3:**
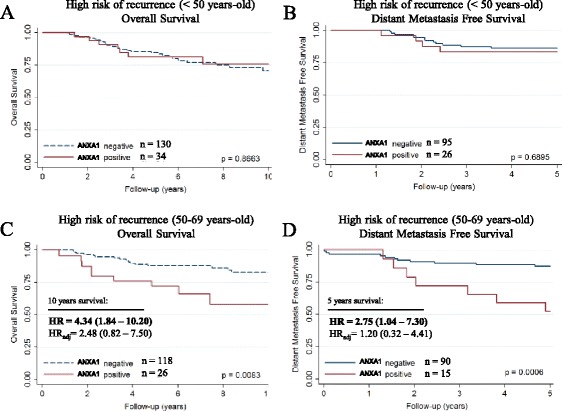
Adjuvant treatment response. Survival curves, crude hazard ratios (HR) and adjusted hazard ratios (HR_adj_) according to ANXA1 expression in patients from BCAC with high risk of recurrence (HER2+ and/or pN+) who received anthracycline-based adjuvant chemotherapy. Overall survival and disease-free survival in (**a**, **b**) patients under 50 years old and (**c**, **d**) patients over 49 and under 70 years old. Hazard ratios were adjusted for: age of diagnosis; tumor grade; tumor size; lymph node metastasis; ER/PR status; and HER2 status. Note: low risk of recurrence was defined as: 35 years old or older; lymph node negative; tumor size ≥2 cm with any grade or tumor size ≥1 cm with tumor grade ≥2; high risk of recurrence was defined as: HER2+ and/or lymph node positive. ANXA1: Annexin A1; BCAC: Breast Cancer Association Consortium; HR: Hazard ratio

Part of these patients (24.2 %) received anthracycline-based adjuvant chemotherapy. In the same high risk group aged 50–69 years, the ANXA1 positive cases presented a suggestive worse outcome after anthracycline-based adjuvant chemotherapy (10-years OS: HR_adj_ = 2.48; 95 % CI = 0.82–7.50; Fig. 3 and BCSS: HR_adj_ = 2.96; 95 % CI = 0.92–9.57; Additional file 7: Figure S4F).

## Discussion

Here, for the first time, the ANXA1 expression in a subset of *BRCA1/2* mutated carriers is described. We found a significantly higher expression of ANXA1 in tumors from familial breast cancer patients with *BRCA1/2* mutations compared with hospital and population-based breast cancer series.

We also found a higher ANXA1 expression in triple negative patients, confirming previous studies [20, 24, 25]; association with poor differentiation grade is also described in other types of cancers [33, 34]. The association between ANXA1 expression and basal markers (EGFR-CK5/6) as shown in this study was also described in our previous work using a smaller cohort of breast cancer patients [24], suggesting that ANXA1 may play a role in EGFR trafficking [8, 35]. Moreover, the higher frequency of ANXA1 expression in younger patients is not surprising since this group develops more often tumors of poor differentiation grade, triple negative status or with basal marker overexpression [36].

The *BRCA1/2* mutated patients belong to a group that already contains a high number of triple negative and basal-like breast cancers [37], but here we showed that triple negative tumors in *BRCA1/2* mutated carriers are even more highly expressing ANXA1 than triple negative patients in the BCAC cohort (Fig. 1). Perhaps such differences may involve the p53 expression, which was higher in the ANXA1 positive tumors (Additional file 6: Table S3). The tumor suppressor gene *TP53* is more commonly altered in *BRCA1/2*-related breast cancers, as measured either by IHC or mutation analysis [38]. Indeed, *in vitro* studies in colon cancer cells suggest the existence of a binding site for p53 in the promoter of the *ANXA1* gene, inducing its expression and phosphorylation [39, 40]. Unfortunately, the group of *BRCA1/2* mutated carriers presented here is small, indicating that other studies focused in the ANXA1 expression profile in this group of patients are required.

In the survival analysis, ANXA1 positive tumors were independently associated with OS and BCSS in the first 5 years, but not in years 5 to 10. Findings of OS and BCSS were in line, signaling that a significant proportion of the patients with breast cancer died from it and not from other causes, which is expected especially since this series included a large proportion of relatively young breast cancer patients (Additional file 7: Figure S4). We had observed some indication for time dependency of ANXA1. Including ANXA1 time dependency in the model, we even found a suggestion of better survival in the ANXA1 positive group in the 5- to 10-year period of follow-up (BCSS: HR_adj_ = 0.65; 95 % CI = 0.40–1.03). For ANXA1 gene expression, using KM plotter [41], a similar trend was seen with a worse recurrence-free survival in the first 5 years (HR = 1.15; CI = 1.18–10.29; *P* = 0.03), but not over the whole 10-year period (HR = 1.08; CI = 0.96–1.21; *P* = 0.21).

In our data, specifically patients with HER2+ tumors and ANXA1, overexpression showed a worse outcome, even after 5 years (10-years BCSS: HR_adj_ = 1.70; 95 % CI = 1.17–2.45). This is in line with the finding from Yom et al., who observed a worse recurrence-free survival for ANXA1 positive cases also in lymph node positive and HER2+ patients [25]. Of note, most of the cases in our study were not treated with trastuzumab due to the period that they were diagnosed. Accordingly, the absolute OS was lower than would be expected after breast cancer treatment nowadays. Even so, the worse relative survival seen in our study is still relevant for HER2+ patients though with some caution because in most countries nowadays HER2+ patients are also treated with trastuzumab. Further research is therefore warranted to investigate the potential of ANXA1 as a predictor of trastuzumab resistance.

Of note, we observed some heterogeneity between the BCAC studies for the percentage of annexin A1 positive tumors (Additional file 1: Table S1A). These did not seem to be fully explained by differences in tumor characteristics (data not shown) and since the staining was done centrally there might have been some influence of the age of the tumor material and/or fixation at the time of embedding.

We also observed that ANXA1 overexpression was associated with worse survival in patients with high risk of recurrence in an age-dependent manner, with worst outcome in premenopausal patients, especially in the group that received anthracycline-based adjuvant chemotherapy. Unfortunately, for many cases, information about the type of chemotherapy was missing. Therefore, this might be due to chance and hypothesizing a mechanism for these findings is difficult. However, Ang et al. suggested that ANXA1 can regulate growth arrest induced by high levels of estrogen [42], which is the typical physiological condition in premenopausal women. To our knowledge there are no other studies that evaluated ANXA1 expression and anthracycline-based chemotherapy resistance. ANXA1 overexpression was associated with cisplatin resistance in lung adenocarcinoma [43], radiotherapy and chemotherapy resistance in nasopharyngeal carcinoma [44], worse chemotherapy response after treatment with docetaxel, cisplatin and 5-fluorouracil in oral squamous cell carcinoma [34], and poor response after neoadjuvant treatment with taxotere and carboplatin in triple negative breast cancer [45].

Although ANXA1 has been described to play a role in metastasis formation in breast cancer [46], the exact mechanism remains unknown. ANXA1 being also expressed in normal myoepithelial cells, the loss of ANXA1 expression in breast carcinomas has been described as a stage of malignant transformation [19, 20, 47]. In breast cancer models, ANXA1 has been shown to modulate cell adhesion and motility [23] by TGFβ-mediated EMT-like switch [24] and by matrix metalloproteinase-9 regulation via NF-κB [21, 48], but another study found conflicting evidence [49]. Moreover, as a glucocorticoid-induced protein, ANXA1 might also be able to provide critical interference in the tumor stroma and its microenvironment cross-talk [17]. Altogether, our findings stress the importance of ANXA1 for prognosis and possibly for therapy resistance in breast cancer. We are also the first to show that there is a link between *BRCA1/2* mutations and ANXA1 overexpression.

## Conclusion

We conclude here that ANXA1 expression is associated with tumors with selected well-known poor prognosis characteristics (e.g. poor differentiation grade, triple negative, *BRCA1/2* mutations). Our survival analysis showed that ANXA1 expression in breast tumors might be a biomarker candidate for breast cancer outcome prediction in high risk groups such as HER2+ cases, playing a complex role in chemotherapy resistance. Further studies are needed to elucidate whether ANXA1 is indeed a prognostic factor or may be used to predict chemotherapy response.

## Additional files

Additional file 1: Table S1. Description of the studies involved in the BCAC, the percentage of tumors with ANXA1 expression and the list of ethics committees that approved the studies. Additional file 2: Figure S1. Immunohistochemistry for annexin A1 (ANXA1) expression in breast tumors. Additional file 3: Figure S2. ANXA1 scoring profile. Additional file 4: Table S2. Comparison of the clinical variables of breast cancer patients in the TMA versus those with ANXA1 scores for the BCAC and BRCA1|2 patient series. Additional file 5: Figure S3. Flowchart showing the criteria used for the selection of patients in each analysis. Additional file 6: Table S3. Evaluation of the association between the ANXA1 expression and clinical variables in invasive tumors of BCAC and BRCA1|2 breast cancer patients. Additional file 7: Figure S4. Survival curves, crude hazard ratios (HR) and adjusted hazard ratios (HR_adj_) in patients from BCAC according to ANXA1 expression. Additional file 8: Table S4. Cox proportional regression hazard models according the ANXA1 expression in all invasive breast cancers from the BCAC. Additional file 9: Figure S5. Survival curves, crude hazard ratios (HR) and adjusted (HR_adj_) according to ANXA1 expression in patients eligible for adjuvant chemotherapy in the BCAC.

## Abbreviations

ANXA1: Annexin A1
BCAC: Breast Cancer Association Consortium
BCSS: Breast cancer-specific survival
CC1: Cell Conditioning 1
CFMPB: Core Facility Molecular Pathology and Biobanking
ChaK1: Channel kinase 1
CI: Confidence interval
DMFS: Distant metastasis-free survival
EGFR: Epidermal growth factor receptor
EMT: Epithelial-mesenchymal transition
ER: Estrogen receptor
HR: Hazard ratio
HUCH: Helsinki University Central Hospital
IHC: Immunohistochemistry
IR: Insulin receptor
OR: Odds ratio
OS: Overall survival
PKA: Protein kinase A
PKC: Protein kinase C
PR: Progesterone receptor
TMA: Tissue microarray
TN: Triple negative

## References

[1] Kittaneh M, Montero AJ, Gluck S (2013). Molecular profiling for breast cancer: a comprehensive review. Biomarkers Canc.

[2] D’Acunto CW, Gbelcova H, Festa M, Ruml T (2014). The complex understanding of Annexin A1 phosphorylation. Cell Signal.

[3] Pepinsky RB, Sinclair LK (1986). Epidermal growth factor-dependent phosphorylation of lipocortin. Nature.

[4] Skouteris GG, Schroder CH (1996). The hepatocyte growth factor receptor kinase-mediated phosphorylation of lipocortin-1 transduces the proliferating signal of the hepatocyte growth factor. J Biol Chem.

[5] Dorovkov MV, Ryazanov AG (2004). Phosphorylation of annexin I by TRPM7 channel-kinase. J Biol Chem.

[6] Varticovski L, Chahwala SB, Whitman M, Cantley L, Schindler D, Chow EP (1988). Location of sites in human lipocortin I that are phosphorylated by protein tyrosine kinases and protein kinases A and C. Biochemistry.

[7] McArthur S, Yazid S, Christian H, Sirha R, Flower R, Buckingham J (2009). Annexin A1 regulates hormone exocytosis through a mechanism involving actin reorganization. FASEB J.

[8] Futter CE, White IJ (2007). Annexins and endocytosis. Traffic.

[9] Rescher U, Gerke V (2004). Annexins – unique membrane binding proteins with diverse functions. J Cell Sci.

[10] Mu D, Gao Z, Guo H, Zhou G, Sun B (2013). Sodium butyrate induces growth inhibition and apoptosis in human prostate cancer DU145 cells by up-regulation of the expression of annexin A1. PLoS One.

[11] Bizzarro V, Fontanella B, Franceschelli S, Pirozzi M, Christian H, Parente L (2010). Role of Annexin A1 in mouse myoblast cell differentiation. J Cell Physiol.

[12] Gavins FN, Hickey MJ (2012). Annexin A1 and the regulation of innate and adaptive immunity. Front Immunol.

[13] Hu N, Flaig MJ, Su H, Shou JZ, Roth MJ, Li WJ (2004). Comprehensive characterization of annexin I alterations in esophageal squamous cell carcinoma. Clin Cancer Res.

[14] Guo C, Liu S, Sun MZ (2013). Potential role of ANXA1 in cancer. Future Oncol.

[15] Deng S, Wang J, Hou L, Li J, Chen G, Jing B (2013). Annexin A1, A2, A4 and A5 play important roles in breast cancer, pancreatic cancer and laryngeal carcinoma, alone and/or synergistically. Oncol Lett.

[16] Wang KL, Wu TT, Resetkova E, Wang H, Correa AM, Hofstetter WL (2006). Expression of annexin A1 in esophageal and esophagogastric junction adenocarcinomas: association with poor outcome. Clin Cancer Res.

[17] Yi M, Schnitzer JE (2009). Impaired tumor growth, metastasis, angiogenesis and wound healing in annexin A1-null mice. Proc Natl Acad Sci U S A.

[18] Boudhraa Z, Rondepierre F, Ouchchane L, Kintossou R, Trzeciakiewicz A, Franck F (2014). Annexin A1 in primary tumors promotes melanoma dissemination. Clin Exp Metastasis.

[19] Schwartz-Albiez R, Koretz K, Moller P, Wirl G (1993). Differential expression of annexins I and II in normal and malignant human mammary epithelial cells. Differentiation.

[20] Cao Y, Li Y, Edelweiss M, Arun B, Rosen D, Resetkova E (2008). Loss of annexin A1 expression in breast cancer progression. Appl Immunohistochem Mol Morphol.

[21] Bist P, Leow SC, Phua QH, Shu S, Zhuang Q, Loh WT (2011). Annexin-1 interacts with NEMO and RIP1 to constitutively activate IKK complex and NF-kappaB: implication in breast cancer metastasis. Oncogene.

[22] Khau T, Langenbach SY, Schuliga M, Harris T, Johnstone CN, Anderson RL (2011). Annexin-1 signals mitogen-stimulated breast tumor cell proliferation by activation of the formyl peptide receptors (FPRs) 1 and 2. FASEB J.

[23] Swa HLF, Blackstock WP, Lim LHK, Gunaratne J (2012). Quantitative proteomics profiling of murine mammary gland cells unravels impact of annexin-1 on DNA damage response, cell adhesion, and migration. Mol Cell Proteomics.

[24] de Graauw M, van Miltenburg MH, Schmidt MK, Pont C, Lalai R, Kartopawiro J (2010). Annexin A1 regulates TGF-beta signaling and promotes metastasis formation of basal-like breast cancer cells. Proc Natl Acad Sci U S A.

[25] Yom CK, Han W, Kim SW, Kim HS, Shin HC, Chang JN (2011). Clinical significance of annexin A1 expression in breast cancer. J Breast Cancer.

[26] Breast Cancer Association Consortium (2006). Commonly studied single-nucleotide polymorphisms and breast cancer: results from the Breast Cancer Association Consortium. J Natl Cancer Inst.

[27] Broeks A, Schmidt MK, Sherman ME, Couch FJ, Hopper JL, Dite GS (2011). Low penetrance breast cancer susceptibility loci are associated with specific breast tumor subtypes: findings from the Breast Cancer Association Consortium. Hum Mol Genet.

[28] van den Broek AJ, Broeks A, Horlings HM, Canisius SV, Braaf LM, Langerod A (2011). Association of the germline TP53 R72P and MDM2 SNP309 variants with breast cancer survival in specific breast tumor subgroups. Breast Cancer Res Treat.

[29] Vahteristo P, Eerola H, Tamminen A, Blomqvist C, Nevanlinna H (2001). A probability model for predicting *BRCA1* and *BRCA2* mutations in breast and breast-ovarian cancer families. Br J Cancer.

[30] Aaltonen K, Blomqvist C, Amini RM, Eerola H, Aittomaki K, Heikkila P (2008). Familial breast cancers without mutations in *BRCA1* or *BRCA2* have low cyclin E and high cyclin D1 in contrast to cancers in *BRCA* mutation carriers. Clin Cancer Res.

[31] Azzato EM, Greenberg D, Shah M, Blows F, Driver KE, Caporaso NE (2009). Prevalent cases in observational studies of cancer survival: do they bias hazard ratio estimates?. Br J Cancer.

[32] Early Breast Cancer Trialists’ Collaborative Group (2005). Effects of chemotherapy and hormonal therapy for early breast cancer on recurrence and 15-year survival: an overview of the randomised trials. Lancet.

[33] Schittenhelm J, Trautmann K, Tabatabai G, Hermann C, Meyermann R, Beschorner R (2009). Comparative analysis of annexin-1 in neuroepithelial tumors shows altered expression with the grade of malignancy but is not associated with survival. Mod Pathol.

[34] Zhu DW, Liu Y, Yang X, Yang CZ, Ma J, Yang X (2013). Low Annexin A1 expression predicts benefit from induction chemotherapy in oral cancer patients with moderate or poor pathologic differentiation grade. BMC Cancer.

[35] Poeter M, Radke S, Koese M, Hessner F, Hegemann A, Musiol A (1833). Disruption of the annexin A1/S100A11 complex increases the migration and clonogenic growth by dysregulating epithelial growth factor (EGF) signaling. Biochim Biophys Acta.

[36] Reis-Filho JS, Tutt AN (2008). Triple negative tumours: a critical review. Histopathology.

[37] Network CGA (2012). Comprehensive molecular portraits of human breast tumours. Nature.

[38] Chappuis PO, Nethercot V, Foulkes WD (2000). Clinico-pathological characteristics of *BRCA1*- and *BRCA2*-related breast cancer. Semin Surg Oncol.

[39] Lecona E, Barrasa JI, Olmo N, Llorente B, Turnay J, Lizarbe MA (2008). Upregulation of annexin A1 expression by butyrate in human colon adenocarcinoma cells: role of p53, NF-Y, and p38 mitogen-activated protein kinase. Mol Cell Biol.

[40] Rahman-Roblick R, Hellman U, Becker S, Bader FG, Auer G, Wiman KG (2008). Proteomic identification of p53-dependent protein phosphorylation. Oncogene.

[41] Gyorffy B, Surowiak P, Budczies J, Lanczky A (2013). Online survival analysis software to assess the prognostic value of biomarkers using transcriptomic data in non-small-cell lung cancer. PLoS One.

[42] Ang EZ, Nguyen HT, Sim HL, Putti TC, Lim LH (2009). Annexin-1 regulates growth arrest induced by high levels of estrogen in MCF-7 breast cancer cells. Mol Cancer Res.

[43] Wang C, Xiao Q, Li YW, Zhao C, Jia N, Li RL (2014). Regulatory mechanisms of annexin-induced chemotherapy resistance in cisplatin resistant lung adenocarcinoma. Asian Pac J Cancer Prev.

[44] Zeng GQ, Cheng AL, Tang J, Li GQ, Li MX, Qu JQ (2013). Annexin A1: a new biomarker for predicting nasopharyngeal carcinoma response to radiotherapy. Med Hypotheses.

[45] He J, Whelan SA, Lu M, Shen D, Chung DU, Saxton RE (2011). Proteomic-based biosignatures in breast cancer classification and prediction of therapeutic response. Int J Proteomics.

[46] Wang LP, Bi J, Yao C, Xu XD, Li XX, Wang SM (2010). Annexin A1 expression and its prognostic significance in human breast cancer. Neoplasma.

[47] Shen D, Nooraie F, Elshimali Y, Lonsberry V, He J, Bose S (2006). Decreased expression of annexin A1 is correlated with breast cancer development and progression as determined by a tissue microarray analysis. Hum Pathol.

[48] Kang H, Ko J, Jang SW (2012). The role of annexin A1 in expression of matrix metalloproteinase-9 and invasion of breast cancer cells. Biochem Biophys Res Commun.

[49] Maschler S, Gebeshuber CA, Wiedemann EM, Alacakaptan M, Schreiber M, Custic I (2010). Annexin A1 attenuates EMT and metastatic potential in breast cancer. EMBO Mol Med.

