# Supplementary material for: Annexin A1 expression in a pooled breast cancer series: association with tumor subtypes and prognosis
Source: BMC Med. 2015 Jul 2;13:156. doi: 10.1186/s12916-015-0392-6 (PMC4489114; doi:10.1186/s12916-015-0392-6)
Supplement: Additional file 1: Table S1. — Description of the studies involved in the BCAC, the percentage of tumors with ANXA1 expression and the list of ethics committees that approved the studies. [file 12916_2015_392_MOESM1_ESM.ppt]

## Slide 1
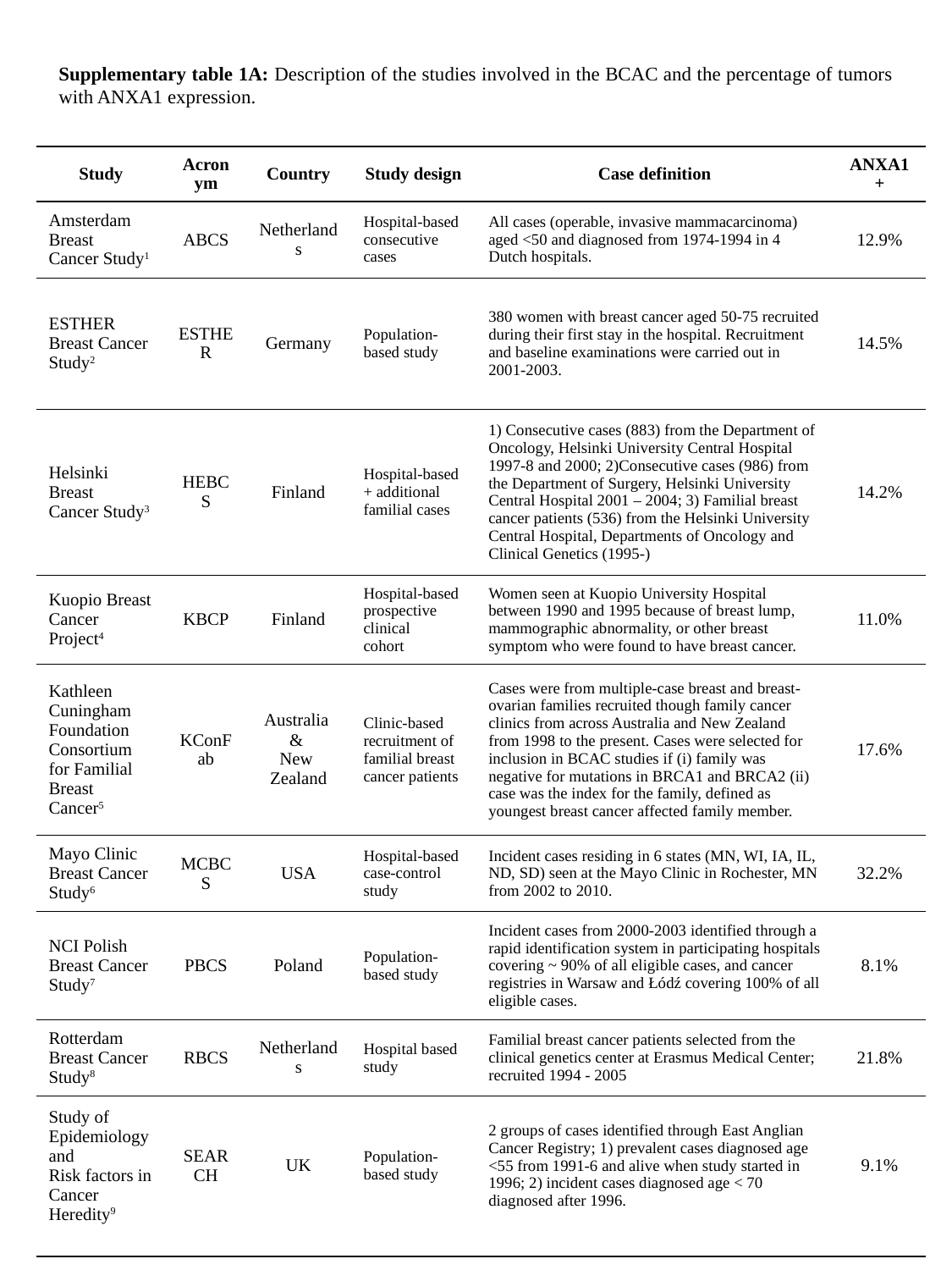

Supplementary table 1A: Description of the studies involved in the BCAC and the percentage of tumors with ANXA1 expression.
| | | | | | |
| --- | --- | --- | --- | --- | --- |
| Study | Acronym | Country | Study design | Case definition | ANXA1+ |
| Amsterdam Breast Cancer Study1 | ABCS | Netherlands | Hospital-based consecutive cases | All cases (operable, invasive mammacarcinoma) aged <50 and diagnosed from 1974-1994 in 4 Dutch hospitals. | 12.9% |
| ESTHER Breast Cancer Study2 | ESTHER | Germany | Population-based study | 380 women with breast cancer aged 50-75 recruited during their first stay in the hospital. Recruitment and baseline examinations were carried out in 2001-2003. | 14.5% |
| Helsinki Breast Cancer Study3 | HEBCS | Finland | Hospital-based + additional familial cases | 1) Consecutive cases (883) from the Department of Oncology, Helsinki University Central Hospital 1997-8 and 2000; 2)Consecutive cases (986) from the Department of Surgery, Helsinki University Central Hospital 2001 – 2004; 3) Familial breast cancer patients (536) from the Helsinki University Central Hospital, Departments of Oncology and Clinical Genetics (1995-) | 14.2% |
| Kuopio Breast Cancer Project4 | KBCP | Finland | Hospital-based prospective clinical cohort | Women seen at Kuopio University Hospital between 1990 and 1995 because of breast lump, mammographic abnormality, or other breast symptom who were found to have breast cancer. | 11.0% |
| Kathleen Cuningham Foundation Consortium for Familial Breast Cancer5 | KConFab | Australia & New Zealand | Clinic-based recruitment of familial breast cancer patients | Cases were from multiple-case breast and breast-ovarian families recruited though family cancer clinics from across Australia and New Zealand from 1998 to the present. Cases were selected for inclusion in BCAC studies if (i) family was negative for mutations in BRCA1 and BRCA2 (ii) case was the index for the family, defined as youngest breast cancer affected family member. | 17.6% |
| Mayo Clinic Breast Cancer Study6 | MCBCS | USA | Hospital-based case-control study | Incident cases residing in 6 states (MN, WI, IA, IL, ND, SD) seen at the Mayo Clinic in Rochester, MN from 2002 to 2010. | 32.2% |
| NCI Polish Breast Cancer Study7 | PBCS | Poland | Population-based study | Incident cases from 2000-2003 identified through a rapid identification system in participating hospitals covering ~ 90% of all eligible cases, and cancer registries in Warsaw and Łódź covering 100% of all eligible cases. | 8.1% |
| Rotterdam Breast Cancer Study8 | RBCS | Netherlands | Hospital based study | Familial breast cancer patients selected from the clinical genetics center at Erasmus Medical Center; recruited 1994 - 2005 | 21.8% |
| Study of Epidemiology and Risk factors in Cancer Heredity9 | SEARCH | UK | Population-based study | 2 groups of cases identified through East Anglian Cancer Registry; 1) prevalent cases diagnosed age <55 from 1991-6 and alive when study started in 1996; 2) incident cases diagnosed age < 70 diagnosed after 1996. | 9.1% |
| | | | | | |
| | | | | | |

## Slide 2
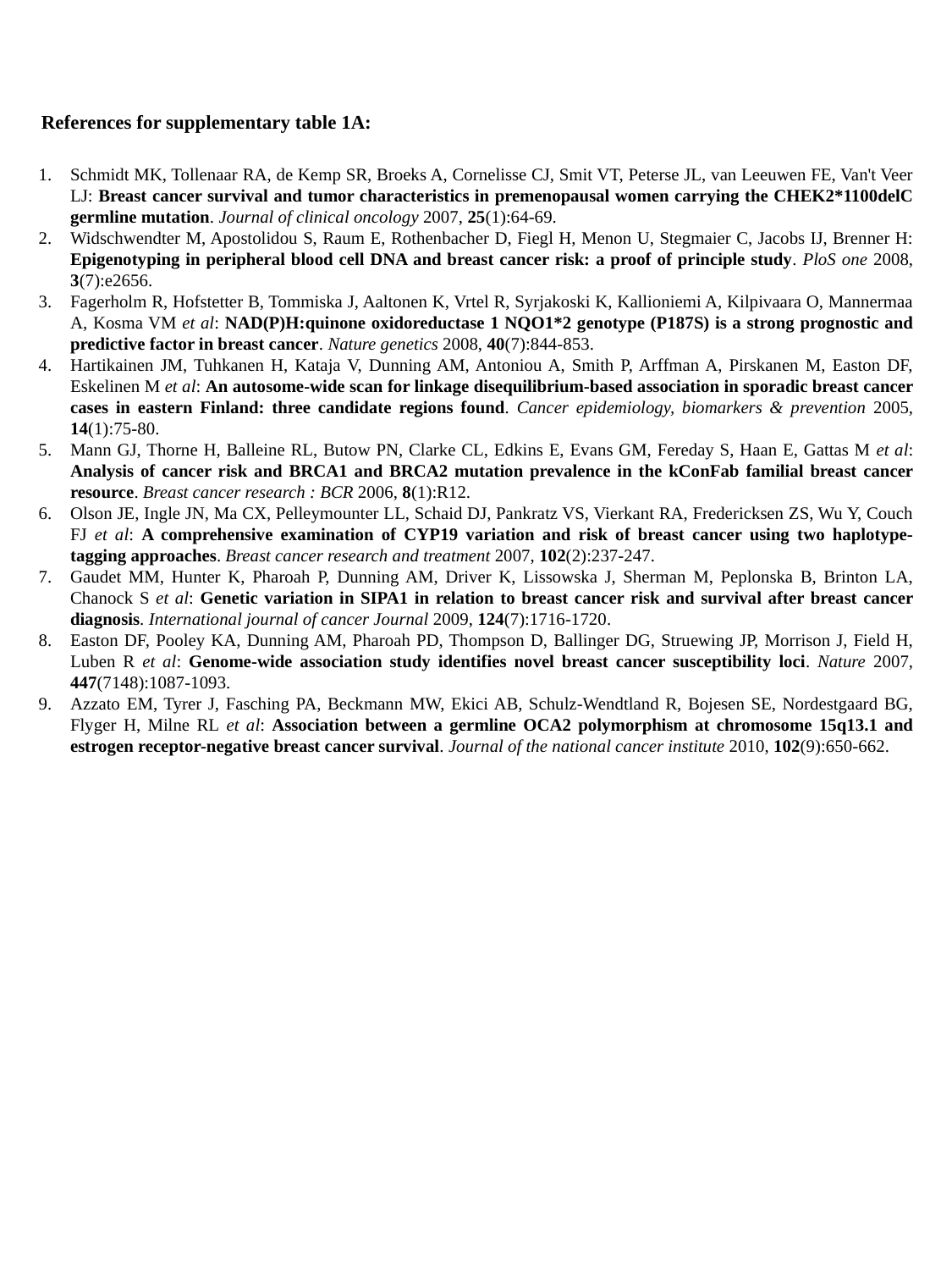

References for supplementary table 1A:
Schmidt MK, Tollenaar RA, de Kemp SR, Broeks A, Cornelisse CJ, Smit VT, Peterse JL, van Leeuwen FE, Van't Veer LJ: Breast cancer survival and tumor characteristics in premenopausal women carrying the CHEK2*1100delC germline mutation. Journal of clinical oncology 2007, 25(1):64-69.
Widschwendter M, Apostolidou S, Raum E, Rothenbacher D, Fiegl H, Menon U, Stegmaier C, Jacobs IJ, Brenner H: Epigenotyping in peripheral blood cell DNA and breast cancer risk: a proof of principle study. PloS one 2008, 3(7):e2656.
Fagerholm R, Hofstetter B, Tommiska J, Aaltonen K, Vrtel R, Syrjakoski K, Kallioniemi A, Kilpivaara O, Mannermaa A, Kosma VM et al: NAD(P)H:quinone oxidoreductase 1 NQO1*2 genotype (P187S) is a strong prognostic and predictive factor in breast cancer. Nature genetics 2008, 40(7):844-853.
Hartikainen JM, Tuhkanen H, Kataja V, Dunning AM, Antoniou A, Smith P, Arffman A, Pirskanen M, Easton DF, Eskelinen M et al: An autosome-wide scan for linkage disequilibrium-based association in sporadic breast cancer cases in eastern Finland: three candidate regions found. Cancer epidemiology, biomarkers & prevention 2005, 14(1):75-80.
Mann GJ, Thorne H, Balleine RL, Butow PN, Clarke CL, Edkins E, Evans GM, Fereday S, Haan E, Gattas M et al: Analysis of cancer risk and BRCA1 and BRCA2 mutation prevalence in the kConFab familial breast cancer resource. Breast cancer research : BCR 2006, 8(1):R12.
Olson JE, Ingle JN, Ma CX, Pelleymounter LL, Schaid DJ, Pankratz VS, Vierkant RA, Fredericksen ZS, Wu Y, Couch FJ et al: A comprehensive examination of CYP19 variation and risk of breast cancer using two haplotype-tagging approaches. Breast cancer research and treatment 2007, 102(2):237-247.
Gaudet MM, Hunter K, Pharoah P, Dunning AM, Driver K, Lissowska J, Sherman M, Peplonska B, Brinton LA, Chanock S et al: Genetic variation in SIPA1 in relation to breast cancer risk and survival after breast cancer diagnosis. International journal of cancer Journal 2009, 124(7):1716-1720.
Easton DF, Pooley KA, Dunning AM, Pharoah PD, Thompson D, Ballinger DG, Struewing JP, Morrison J, Field H, Luben R et al: Genome-wide association study identifies novel breast cancer susceptibility loci. Nature 2007, 447(7148):1087-1093.
Azzato EM, Tyrer J, Fasching PA, Beckmann MW, Ekici AB, Schulz-Wendtland R, Bojesen SE, Nordestgaard BG, Flyger H, Milne RL et al: Association between a germline OCA2 polymorphism at chromosome 15q13.1 and estrogen receptor-negative breast cancer survival. Journal of the national cancer institute 2010, 102(9):650-662.

## Slide 3
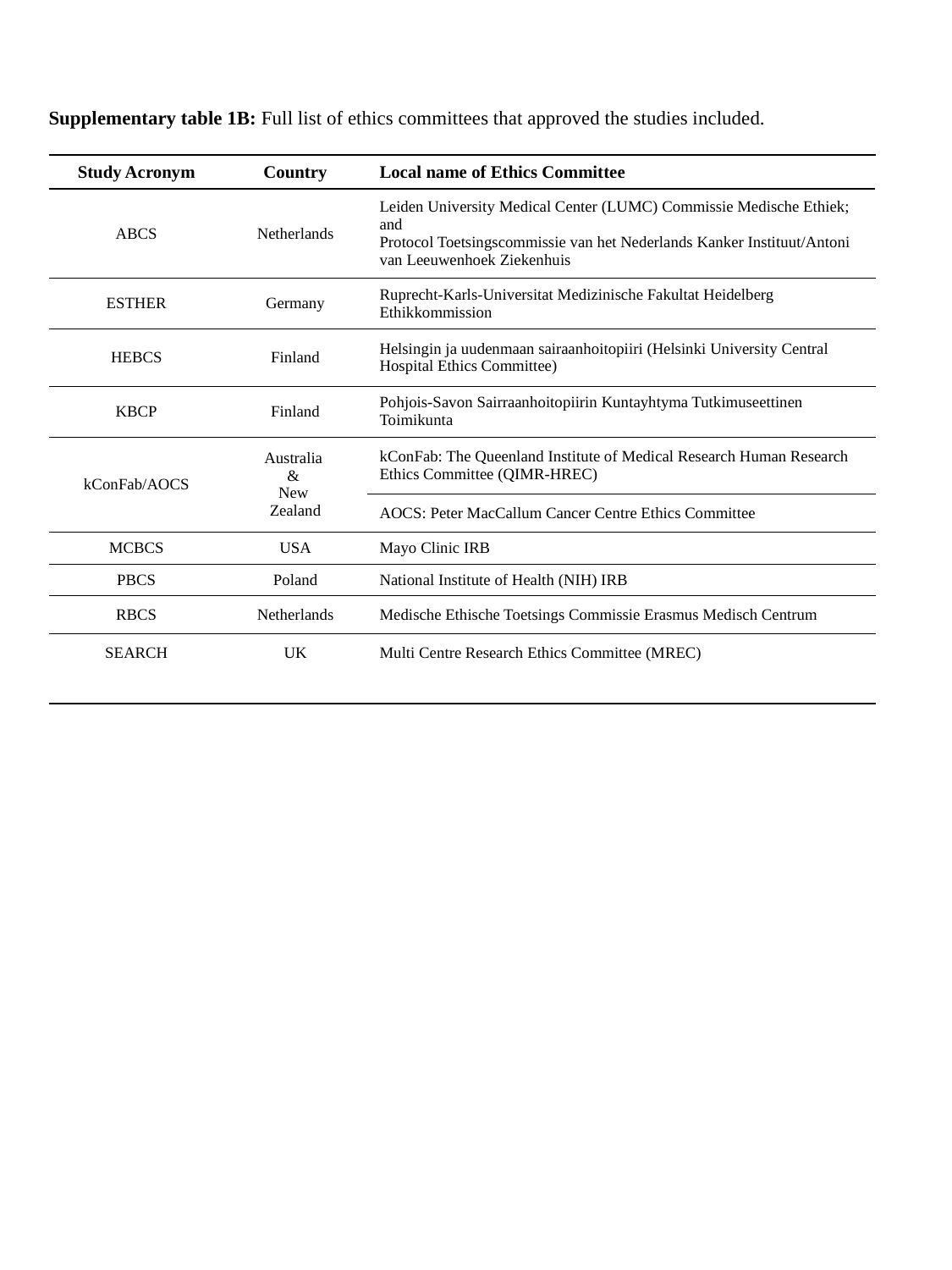

Supplementary table 1B: Full list of ethics committees that approved the studies included.
| Study Acronym | Country | Local name of Ethics Committee |
| --- | --- | --- |
| ABCS | Netherlands | Leiden University Medical Center (LUMC) Commissie Medische Ethiek; and Protocol Toetsingscommissie van het Nederlands Kanker Instituut/Antoni van Leeuwenhoek Ziekenhuis |
| ESTHER | Germany | Ruprecht-Karls-Universitat Medizinische Fakultat Heidelberg Ethikkommission |
| HEBCS | Finland | Helsingin ja uudenmaan sairaanhoitopiiri (Helsinki University Central Hospital Ethics Committee) |
| KBCP | Finland | Pohjois-Savon Sairraanhoitopiirin Kuntayhtyma Tutkimuseettinen Toimikunta |
| kConFab/AOCS | Australia & New Zealand | kConFab: The Queenland Institute of Medical Research Human Research Ethics Committee (QIMR-HREC) |
| | | AOCS: Peter MacCallum Cancer Centre Ethics Committee |
| MCBCS | USA | Mayo Clinic IRB |
| PBCS | Poland | National Institute of Health (NIH) IRB |
| RBCS | Netherlands | Medische Ethische Toetsings Commissie Erasmus Medisch Centrum |
| SEARCH | UK | Multi Centre Research Ethics Committee (MREC) |
| | | |
