# Supplementary material for: Annexin A1 expression in a pooled breast cancer series: association with tumor subtypes and prognosis
Source: BMC Med. 2015 Jul 2;13:156. doi: 10.1186/s12916-015-0392-6 (PMC4489114; doi:10.1186/s12916-015-0392-6)
Supplement: Additional file 2: Figure S1. — Immunohistochemistry for annexin A1 (ANXA1) expression in breast tumors. [file 12916_2015_392_MOESM2_ESM.ppt]

## Slide 1
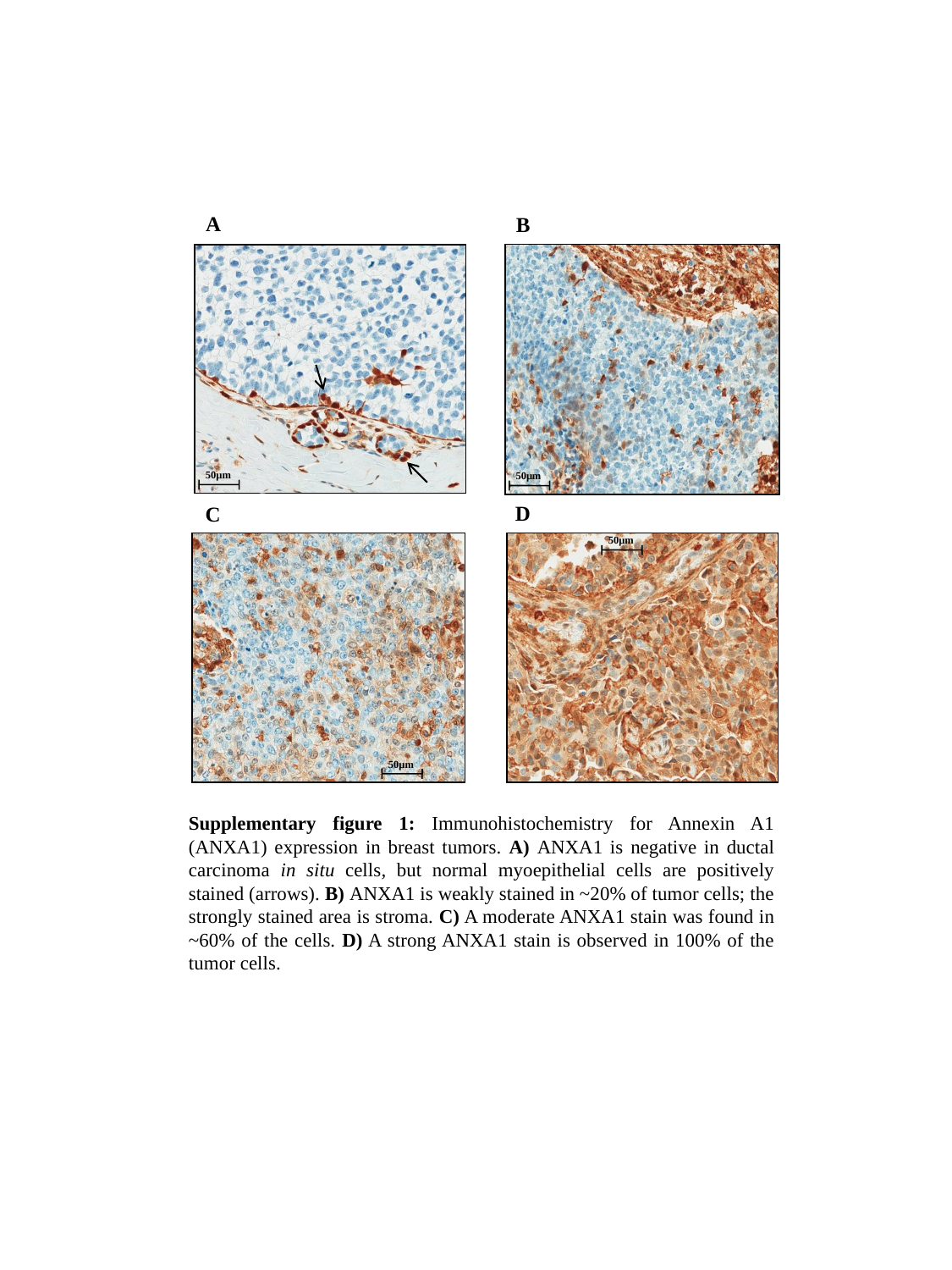

A
B
50μm
50μm
C
D
C
50μm
50μm
Supplementary figure 1: Immunohistochemistry for Annexin A1 (ANXA1) expression in breast tumors. A) ANXA1 is negative in ductal carcinoma in situ cells, but normal myoepithelial cells are positively stained (arrows). B) ANXA1 is weakly stained in ~20% of tumor cells; the strongly stained area is stroma. C) A moderate ANXA1 stain was found in ~60% of the cells. D) A strong ANXA1 stain is observed in 100% of the tumor cells.
