# Supplementary material for: Annexin A1 expression in a pooled breast cancer series: association with tumor subtypes and prognosis
Source: BMC Med. 2015 Jul 2;13:156. doi: 10.1186/s12916-015-0392-6 (PMC4489114; doi:10.1186/s12916-015-0392-6)
Supplement: Additional file 3: Figure S2. — ANXA1 scoring profile. [file 12916_2015_392_MOESM3_ESM.ppt]

## Slide 1
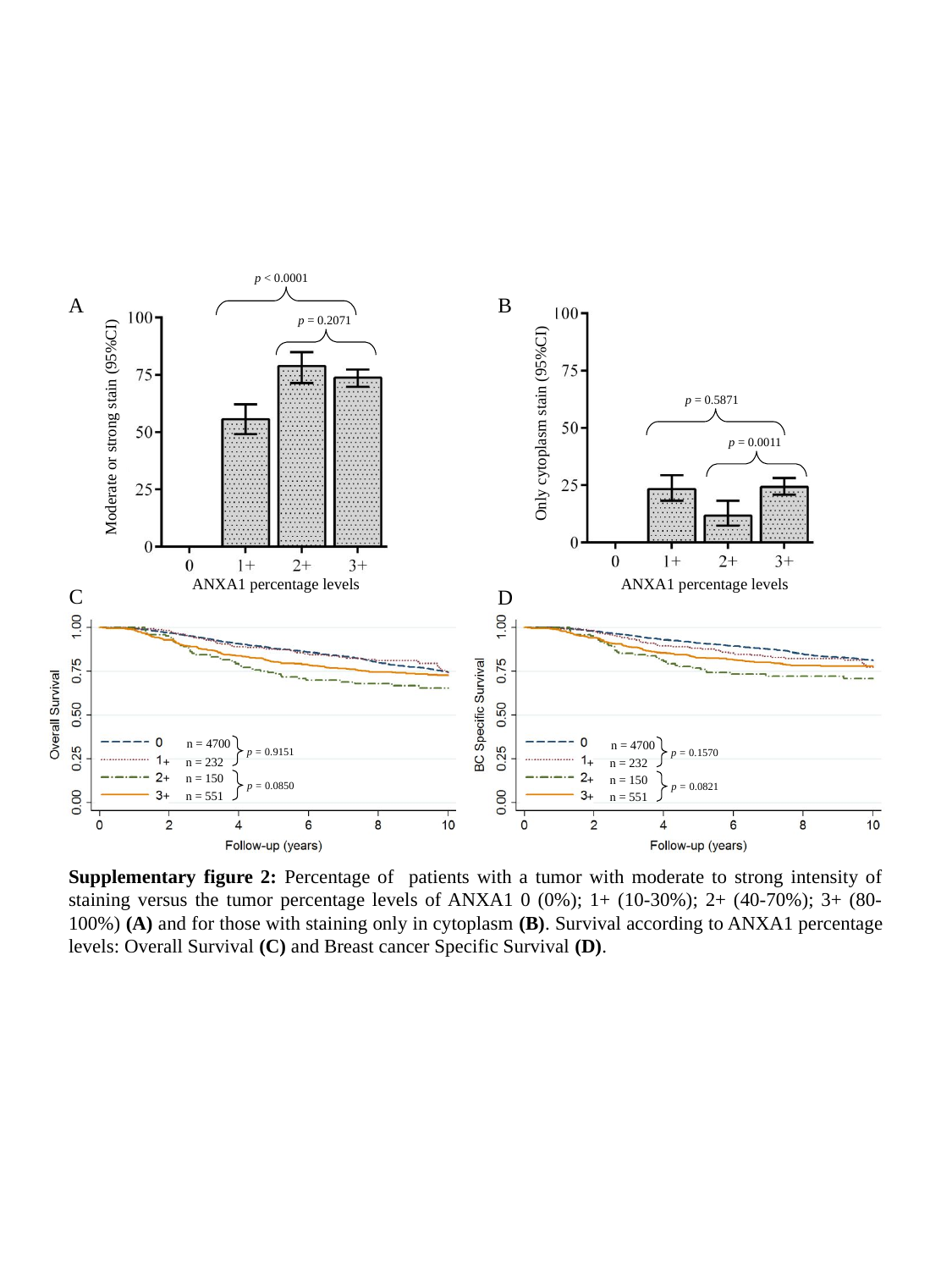

p < 0.0001
A
B
p = 0.2071
p = 0.5871
Only cytoplasm stain (95%CI)
Moderate or strong stain (95%CI)
p = 0.0011
ANXA1 percentage levels
ANXA1 percentage levels
C
D
 n = 4700
 n = 4700
p = 0.9151
p = 0.1570
+ n = 232
+ n = 232
+ n = 150
+ n = 150
p = 0.0850
p = 0.0821
+ n = 551
+ n = 551
Supplementary figure 2: Percentage of patients with a tumor with moderate to strong intensity of staining versus the tumor percentage levels of ANXA1 0 (0%); 1+ (10-30%); 2+ (40-70%); 3+ (80-100%) (A) and for those with staining only in cytoplasm (B). Survival according to ANXA1 percentage levels: Overall Survival (C) and Breast cancer Specific Survival (D).
