# Supplementary material for: Annexin A1 expression in a pooled breast cancer series: association with tumor subtypes and prognosis
Source: BMC Med. 2015 Jul 2;13:156. doi: 10.1186/s12916-015-0392-6 (PMC4489114; doi:10.1186/s12916-015-0392-6)
Supplement: Additional file 4: Table S2. — Comparison of the clinical variables of breast cancer patients in the TMA versus those with ANXA1 scores for the BCAC and BRCA1|2 patient series. [file 12916_2015_392_MOESM4_ESM.ppt]

## Slide 1
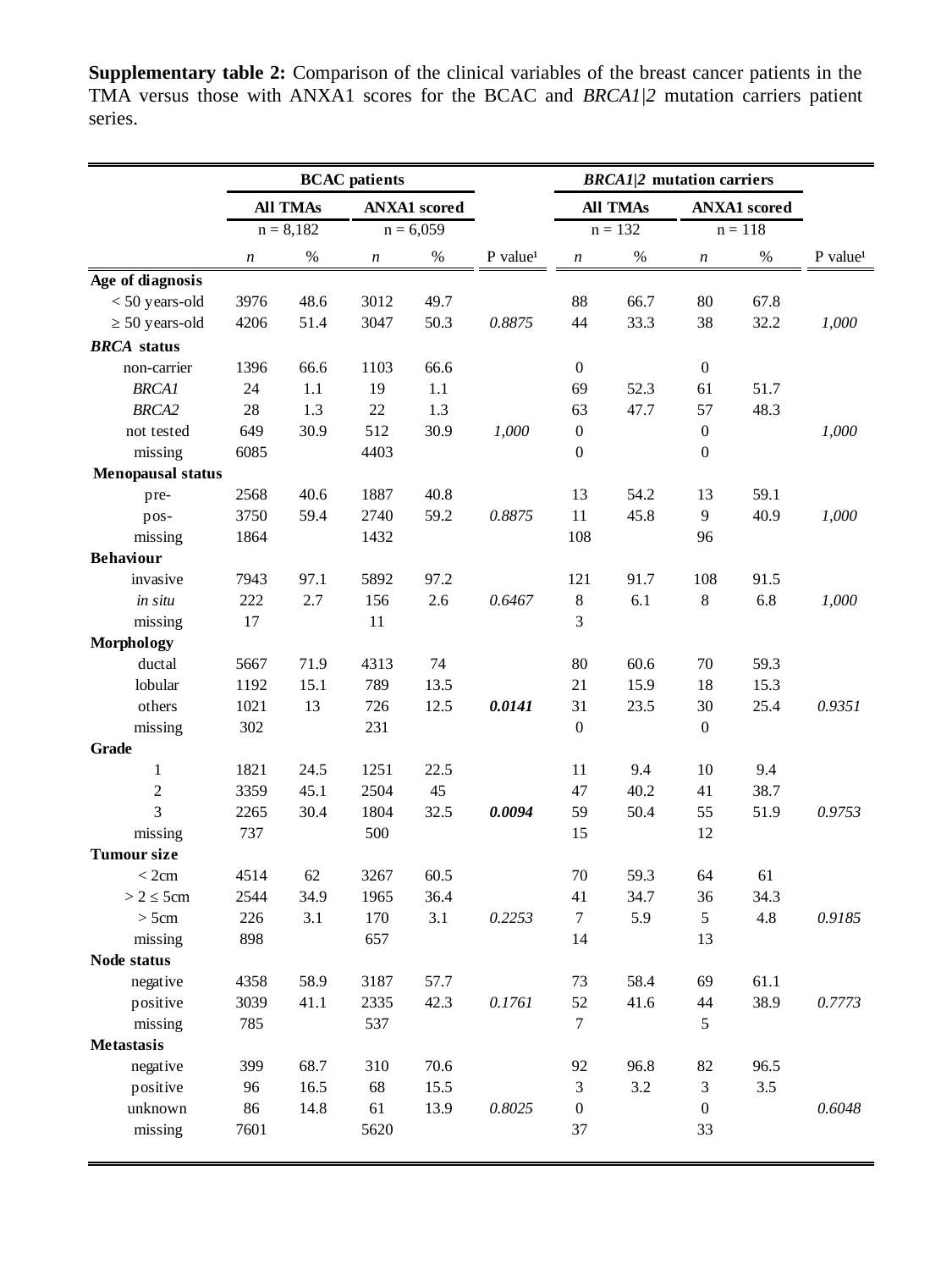

Supplementary table 2: Comparison of the clinical variables of the breast cancer patients in the TMA versus those with ANXA1 scores for the BCAC and BRCA1|2 mutation carriers patient series.

## Slide 2
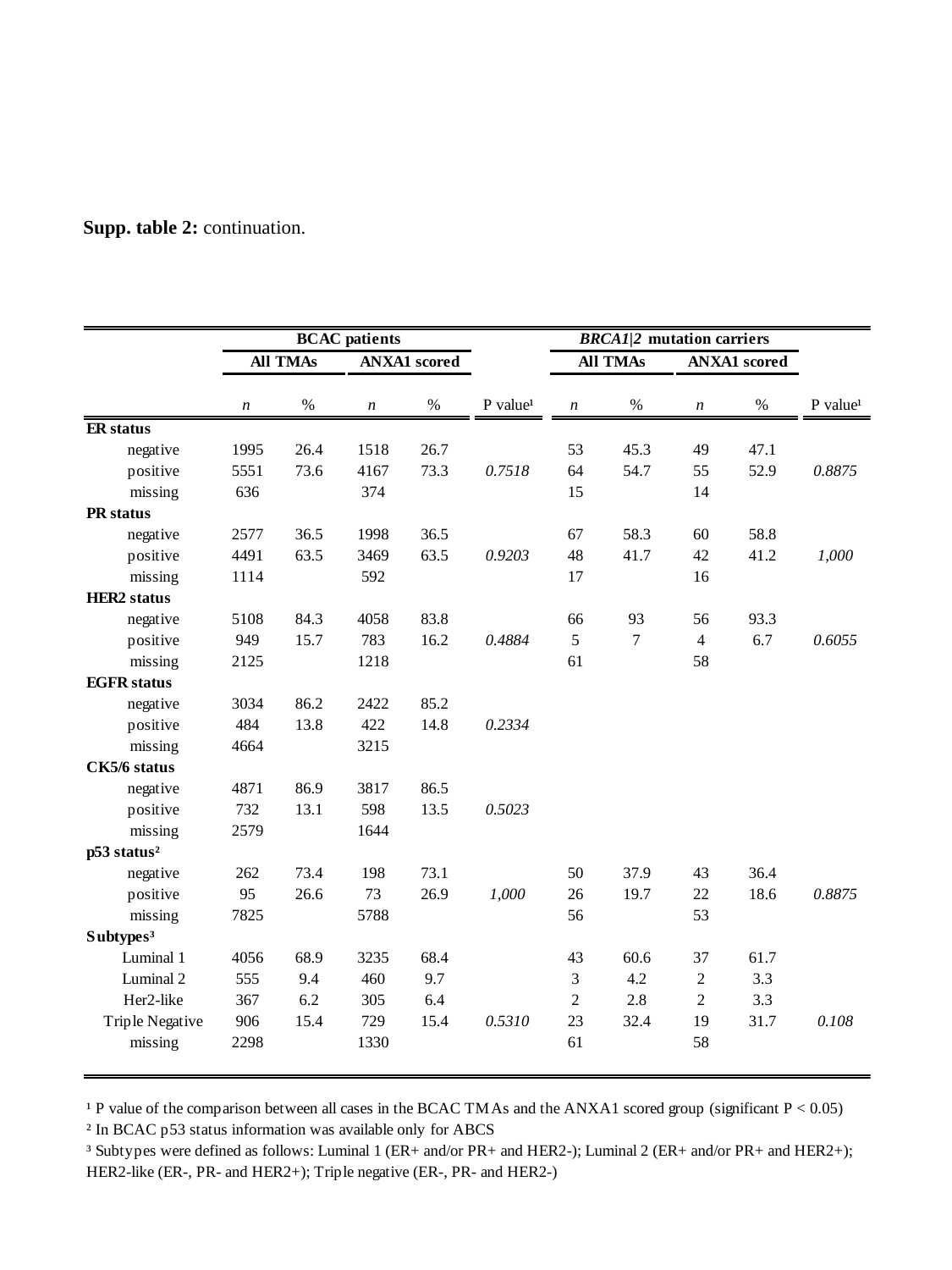

Supp. table 2: continuation.
