# Supplementary material for: Annexin A1 expression in a pooled breast cancer series: association with tumor subtypes and prognosis
Source: BMC Med. 2015 Jul 2;13:156. doi: 10.1186/s12916-015-0392-6 (PMC4489114; doi:10.1186/s12916-015-0392-6)
Supplement: Additional file 5: Figure S3. — Flowchart showing the criteria used for the selection of patients in each analysis. [file 12916_2015_392_MOESM5_ESM.ppt]

## Slide 1
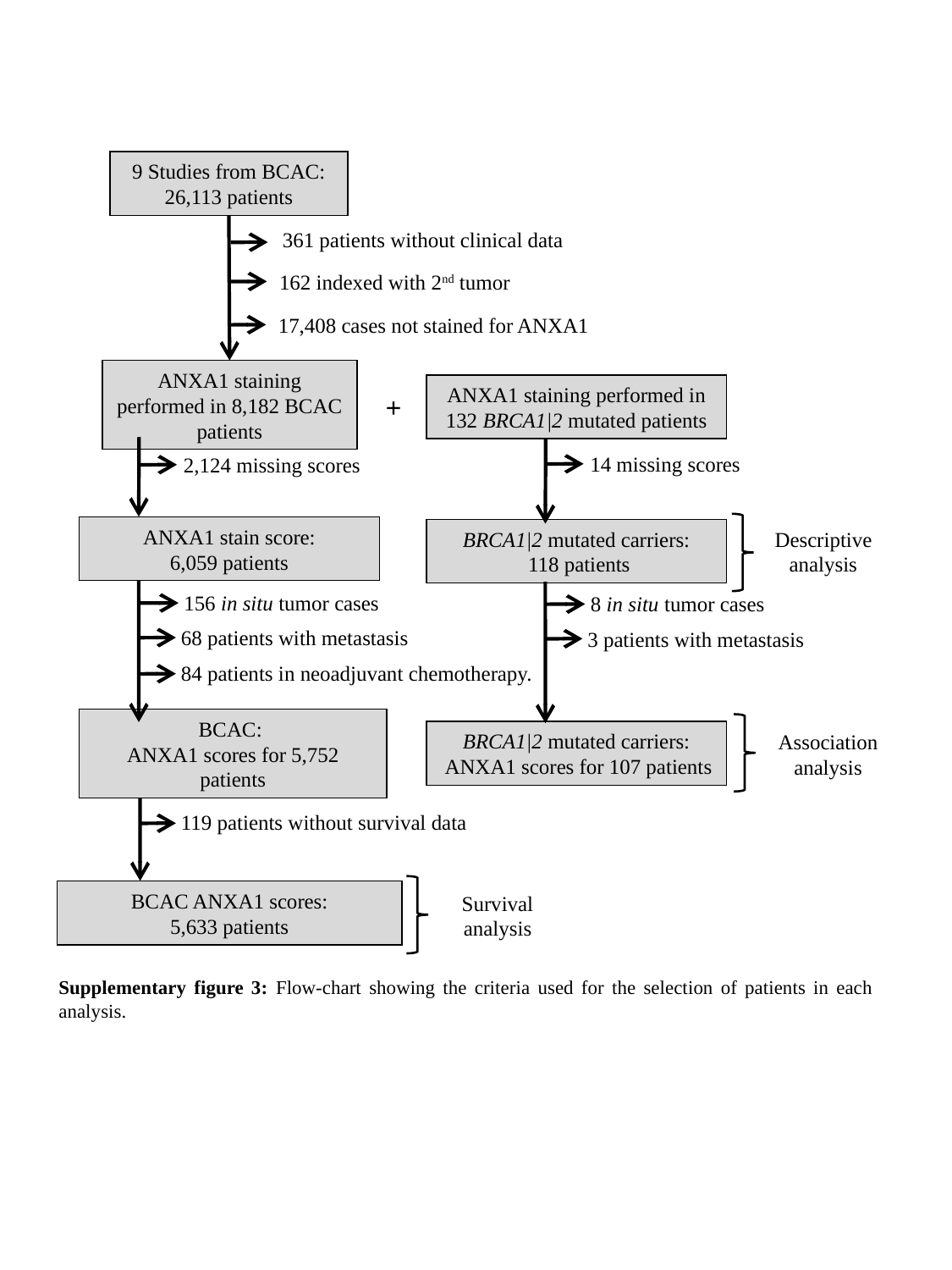

9 Studies from BCAC:
26,113 patients
361 patients without clinical data
 162 indexed with 2nd tumor
 17,408 cases not stained for ANXA1
ANXA1 staining performed in 8,182 BCAC patients
ANXA1 staining performed in 132 BRCA1|2 mutated patients
 +
 14 missing scores
 2,124 missing scores
ANXA1 stain score:
6,059 patients
BRCA1|2 mutated carriers:
 118 patients
Descriptive analysis
156 in situ tumor cases
8 in situ tumor cases
68 patients with metastasis
3 patients with metastasis
84 patients in neoadjuvant chemotherapy.
BCAC:
ANXA1 scores for 5,752 patients
BRCA1|2 mutated carriers:
 ANXA1 scores for 107 patients
Association analysis
119 patients without survival data
BCAC ANXA1 scores:
5,633 patients
Survival analysis
Supplementary figure 3: Flow-chart showing the criteria used for the selection of patients in each analysis.
