# Supplementary material for: Annexin A1 expression in a pooled breast cancer series: association with tumor subtypes and prognosis
Source: BMC Med. 2015 Jul 2;13:156. doi: 10.1186/s12916-015-0392-6 (PMC4489114; doi:10.1186/s12916-015-0392-6)
Supplement: Additional file 6: Table S3. — Evaluation of the association between the ANXA1 expression and clinical variables in invasive tumors of BCAC and BRCA1|2 breast cancer patients. [file 12916_2015_392_MOESM6_ESM.ppt]

## Slide 1
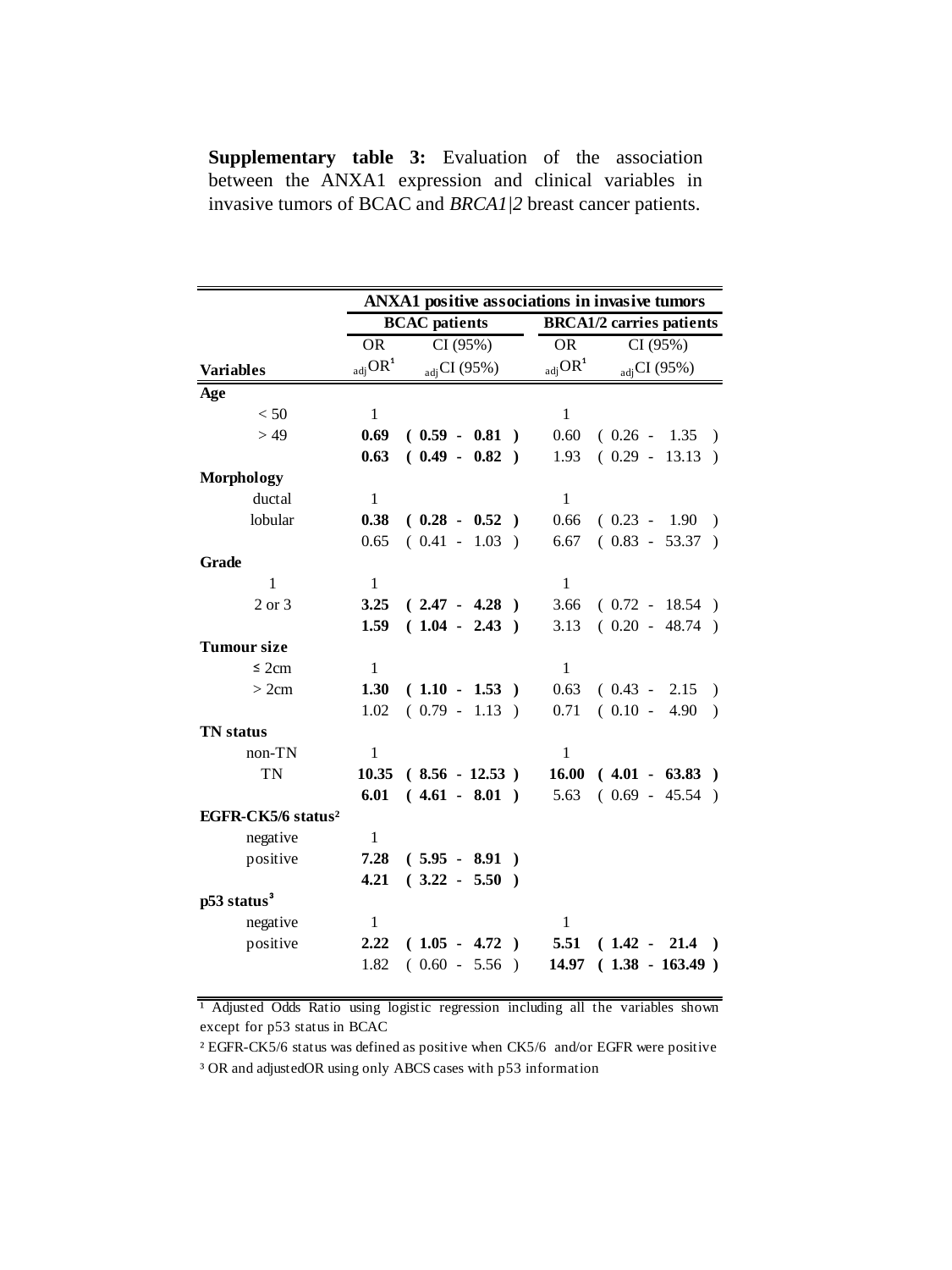

Supplementary table 3: Evaluation of the association between the ANXA1 expression and clinical variables in invasive tumors of BCAC and BRCA1|2 breast cancer patients.
