# Supplementary material for: Annexin A1 expression in a pooled breast cancer series: association with tumor subtypes and prognosis
Source: BMC Med. 2015 Jul 2;13:156. doi: 10.1186/s12916-015-0392-6 (PMC4489114; doi:10.1186/s12916-015-0392-6)
Supplement: Additional file 7: Figure S4. — Survival curves, crude hazard ratios (HR) and adjusted hazard ratios (HRadj) in patients from BCAC according to ANXA1 expression. [file 12916_2015_392_MOESM7_ESM.ppt]

## Slide 1
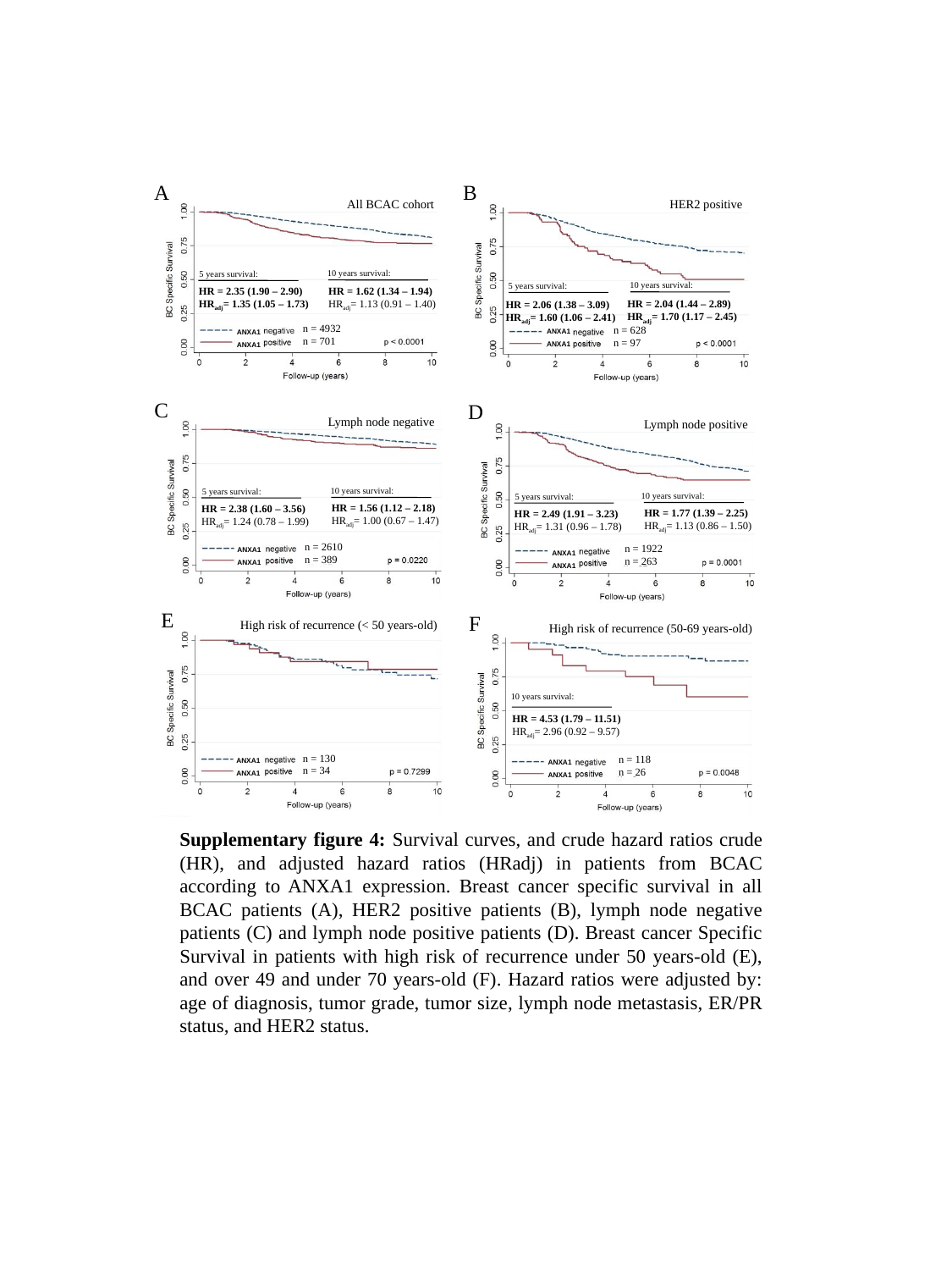

A
B
All BCAC cohort
HER2 positive
10 years survival:
5 years survival:
10 years survival:
5 years survival:
HR = 1.62 (1.34 – 1.94)
HRadj= 1.13 (0.91 – 1.40)
HR = 2.35 (1.90 – 2.90)
HRadj= 1.35 (1.05 – 1.73)
HR = 2.04 (1.44 – 2.89)
HRadj= 1.70 (1.17 – 2.45)
HR = 2.06 (1.38 – 3.09)
HRadj= 1.60 (1.06 – 2.41)
n = 4932
n = 701
ANXA1
ANXA1
n = 628
n = 97
ANXA1
ANXA1
C
D
Lymph node negative
Lymph node positive
10 years survival:
5 years survival:
10 years survival:
5 years survival:
HR = 1.56 (1.12 – 2.18)
HRadj= 1.00 (0.67 – 1.47)
HR = 2.38 (1.60 – 3.56)
HRadj= 1.24 (0.78 – 1.99)
HR = 1.77 (1.39 – 2.25)
HRadj= 1.13 (0.86 – 1.50)
HR = 2.49 (1.91 – 3.23)
HRadj= 1.31 (0.96 – 1.78)
n = 2610
n = 389
ANXA1
ANXA1
n = 1922
n = 263
ANXA1
ANXA1
E
F
High risk of recurrence (< 50 years-old)
High risk of recurrence (50-69 years-old)
10 years survival:
HR = 4.53 (1.79 – 11.51)
HRadj= 2.96 (0.92 – 9.57)
n = 130
n = 34
ANXA1
ANXA1
n = 118
n = 26
ANXA1
ANXA1
Supplementary figure 4: Survival curves, and crude hazard ratios crude (HR), and adjusted hazard ratios (HRadj) in patients from BCAC according to ANXA1 expression. Breast cancer specific survival in all BCAC patients (A), HER2 positive patients (B), lymph node negative patients (C) and lymph node positive patients (D). Breast cancer Specific Survival in patients with high risk of recurrence under 50 years-old (E), and over 49 and under 70 years-old (F). Hazard ratios were adjusted by: age of diagnosis, tumor grade, tumor size, lymph node metastasis, ER/PR status, and HER2 status.
