# Supplementary material for: Annexin A1 expression in a pooled breast cancer series: association with tumor subtypes and prognosis
Source: BMC Med. 2015 Jul 2;13:156. doi: 10.1186/s12916-015-0392-6 (PMC4489114; doi:10.1186/s12916-015-0392-6)
Supplement: Additional file 8: Table S4. — Cox proportional regression hazard models according the ANXA1 expression in all invasive breast cancers from the BCAC. [file 12916_2015_392_MOESM8_ESM.ppt]

## Slide 1
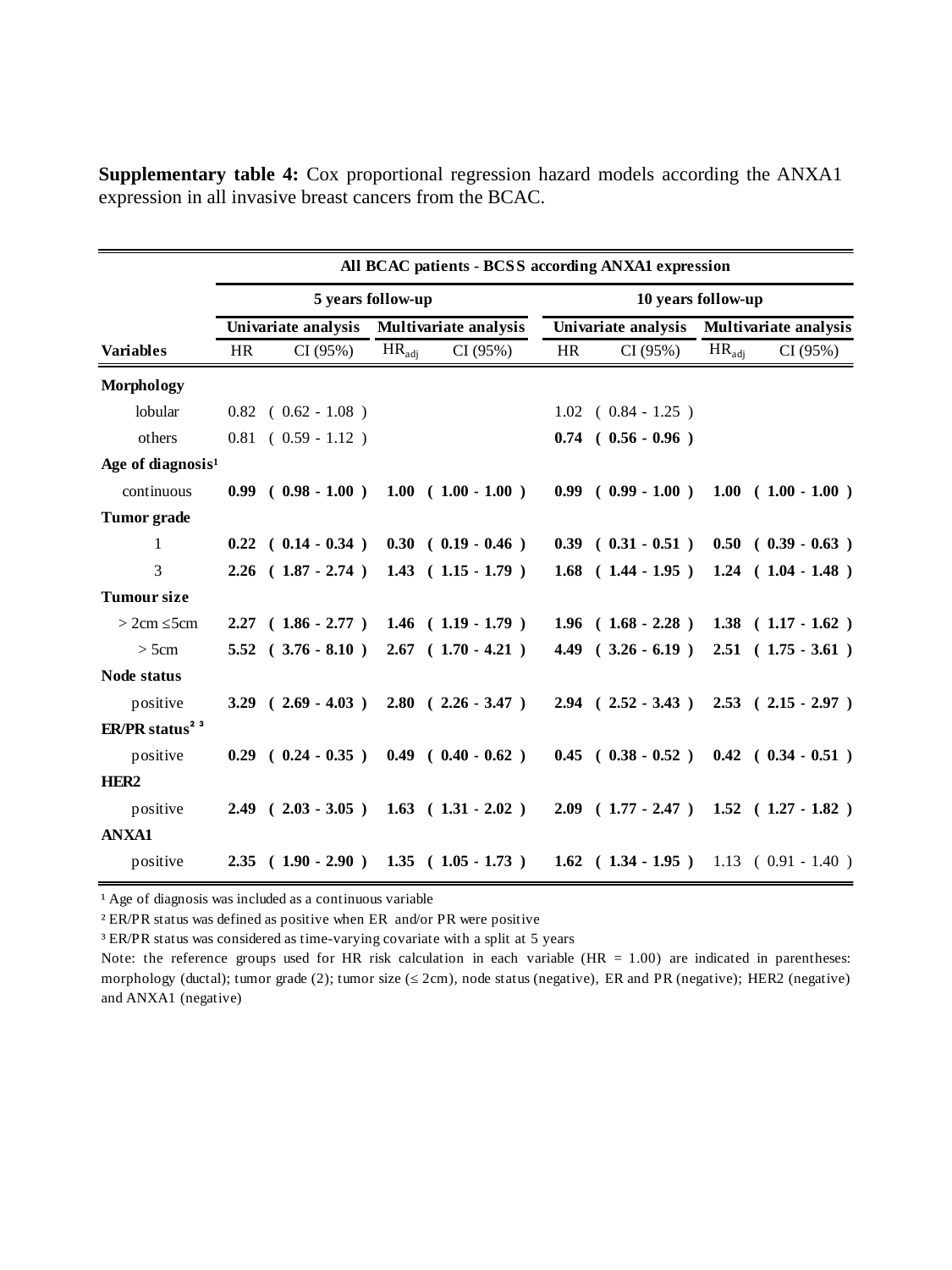

Supplementary table 4: Cox proportional regression hazard models according the ANXA1 expression in all invasive breast cancers from the BCAC.
