# Supplementary material for: Annexin A1 expression in a pooled breast cancer series: association with tumor subtypes and prognosis
Source: BMC Med. 2015 Jul 2;13:156. doi: 10.1186/s12916-015-0392-6 (PMC4489114; doi:10.1186/s12916-015-0392-6)
Supplement: Additional file 9: Figure S5. — Survival curves, crude hazard ratios (HR) and adjusted (HRadj) according to ANXA1 expression in patients eligible for adjuvant chemotherapy in the BCAC. [file 12916_2015_392_MOESM9_ESM.ppt]

## Slide 1
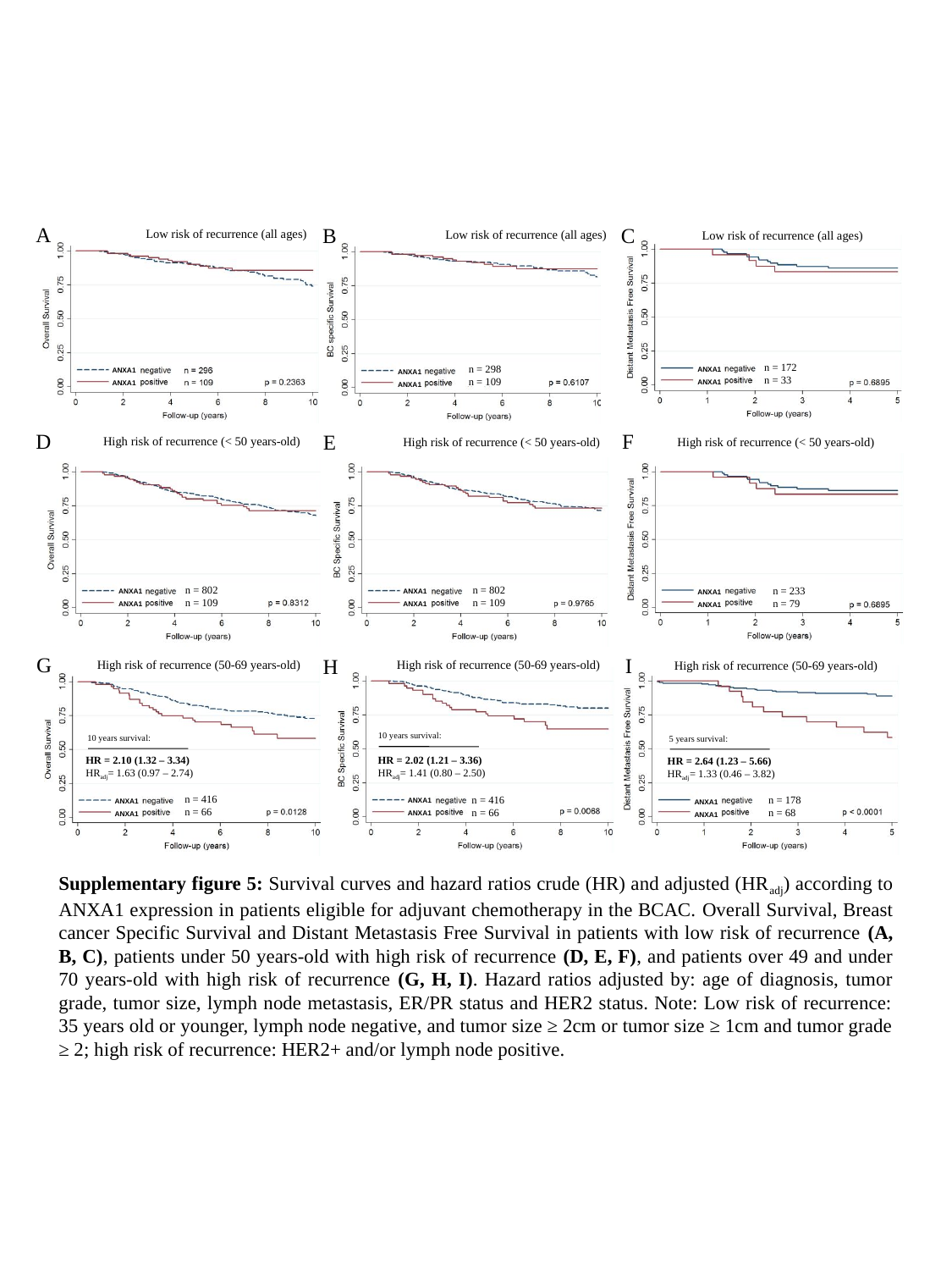

A
C
B
Low risk of recurrence (all ages)
Low risk of recurrence (all ages)
Low risk of recurrence (all ages)
n = 172
n = 33
ANXA1
ANXA1
ANXA1
ANXA1
n = 298
n = 109
ANXA1
ANXA1
D
F
E
High risk of recurrence (< 50 years-old)
High risk of recurrence (< 50 years-old)
High risk of recurrence (< 50 years-old)
ANXA1
ANXA1
ANXA1
ANXA1
n = 802
n = 109
n = 802
n = 109
ANXA1
ANXA1
n = 233
n = 79
G
I
H
High risk of recurrence (50-69 years-old)
High risk of recurrence (50-69 years-old)
High risk of recurrence (50-69 years-old)
10 years survival:
10 years survival:
5 years survival:
HR = 2.02 (1.21 – 3.36)
HRadj= 1.41 (0.80 – 2.50)
HR = 2.10 (1.32 – 3.34)
HRadj= 1.63 (0.97 – 2.74)
HR = 2.64 (1.23 – 5.66)
HRadj= 1.33 (0.46 – 3.82)
ANXA1
ANXA1
ANXA1
ANXA1
n = 416
n = 66
n = 416
n = 66
ANXA1
ANXA1
n = 178
n = 68
Supplementary figure 5: Survival curves and hazard ratios crude (HR) and adjusted (HRadj) according to ANXA1 expression in patients eligible for adjuvant chemotherapy in the BCAC. Overall Survival, Breast cancer Specific Survival and Distant Metastasis Free Survival in patients with low risk of recurrence (A, B, C), patients under 50 years-old with high risk of recurrence (D, E, F), and patients over 49 and under 70 years-old with high risk of recurrence (G, H, I). Hazard ratios adjusted by: age of diagnosis, tumor grade, tumor size, lymph node metastasis, ER/PR status and HER2 status. Note: Low risk of recurrence: 35 years old or younger, lymph node negative, and tumor size ≥ 2cm or tumor size ≥ 1cm and tumor grade ≥ 2; high risk of recurrence: HER2+ and/or lymph node positive.
